# A MOF/DNA luminescent sensing platform for detection of potential COVID-19 biomarkers and drugs†

**DOI:** 10.1039/d3sc00106g

**Published:** 2023-04-19

**Authors:** Xinrui Wang, Gilles Clavier, Yan Zhang, Kamal Batra, Nanan Xiao, Guillaume Maurin, Bin Ding, Antoine Tissot, Christian Serre

**Affiliations:** a Institut des Matériaux Poreux de Paris, Ecole Normale Supérieure, ESPCI Paris, CNRS, PSL University 75005 Paris France antoine.tissot@ens.psl.eu christian.serre@ens.psl.eu; b Université Paris-Saclay, ENS Paris-Saclay, CNRS, PPSM 91190 Gif-sur-Yvette France; c Key Laboratory of Advanced Energy Materials Chemistry (Ministry of Education), Collaborative Innovation Center of Chemical Science and Engineering (Tianjin), College of Chemistry, Nankai University Tianjin 300071 P. R. China; d ICGM, Univ. Montpellier, CNRS, ENSCM Montpellier 34095 France; e Tianjin Key Laboratory of Structure and Performance for Functional Molecule, College of Chemistry, Tianjin Normal University 393 Binshui West Road Tianjin 300387 P. R. China hxxydb@tjnu.edu.cn

## Abstract

COVID-19 has afflicted people's lives worldwide. Interleukin-6 (IL-6) is an important COVID-19 biomarker in human body fluids that can be used as a reference to monitor COVID-19 in real-time and therefore to reduce the risk of virus transmission. On the other hand, oseltamivir is a potential COVID-19 curing drug, but its overuse easily leads to hazardous side effects, calling for its real time monitoring in body fluids. For these purposes, a new yttrium metal–organic framework (Y-MOF) has been synthesized, in which the 5-(4-(imidazole-1-yl)phenyl)isophthalic linker contains a large aromatic backbone capable of strongly interacting with DNA sequences through π–π stacking interactions, which makes it appealing to build a unique sensor based on DNA functionalized MOFs. The MOF/DNA sequence hybrid luminescent sensing platform presents excellent optical properties associated with a high Förster resonance energy transfer (FRET) efficiency. Furthermore, to construct a dual emission sensing platform, a 5′-carboxylfluorescein (FAM) labeled DNA sequence (S2) with a stem-loop structure that can specifically interact with IL-6 has been associated with the Y-MOF. The resulting Y-MOF@S2 exhibits an efficient ratiometric detection of IL-6 in human body fluids with an extremely high *K*_sv_ value 4.3 × 10^8^ M^−1^ and a low detection limit (LOD) of 70 pM. Finally, the Y-MOF@S2@IL-6 hybrid platform allows the detection of oseltamivir with high sensitivity (*K*_sv_ value is as high as 5.6 × 10^5^ M^−1^ and LOD is 54 nM), due to the fact that oseltamivir can disconnect the loop stem structure constructed by S2, leading to a strong quenching effect towards Y-MOF@S2@IL-6. The nature of the interactions between oseltamivir and Y-MOF has been elucidated using density functional theory calculations while the sensing mechanism for the dual detection of IL-6 and oseltamivir has been deciphered based on luminescence lifetime tests and confocal laser scanning microscopy.

## Abstract 2

A new yttrium metal–organic framework has been synthesized and functionalized with a specific DNA sequence to perform the selective ratiometric luminescence sensing of IL-6 and oseltamivir that are important COVID-19 biomarker and potential drug.

## Graphical abstract

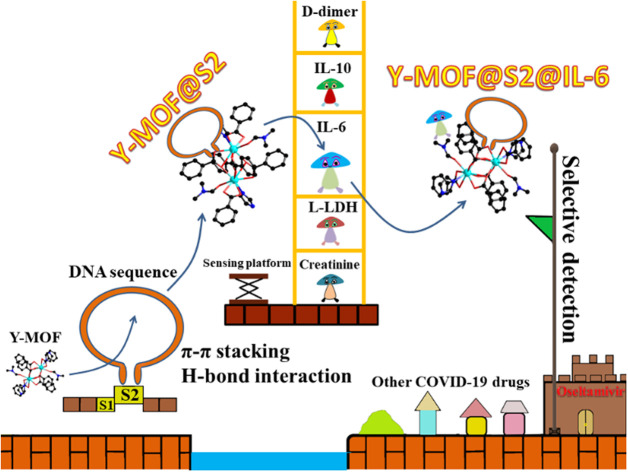

## Introduction

During the past three years, the highly contagious COVID-19 disease has led to a huge death toll and concomitantly has deeply affected our lives. To avoid a further epidemic outbreak and help control the situation, it is crucial to monitor the amount of COVID-19 biomarkers in real-time. Elevated levels of interleukin-6 (IL-6) in human blood are commonly seen in patients suffering from severe COVID-19 illness, which has been confirmed by viral detection in throat samples in both survivors and non-survivors.^1,2^ In addition, oseltamivir is a potential COVID-19 drug that prevents the connection between the mature virus and the host cell, thus decreasing the spread of virus replication.^2^ However, overuse of oseltamivir easily leads to hazardous side effects such as gastrointestinal discomfort, nausea, vomiting and diarrhea, followed by respiratory adverse reactions, including bronchitis and cough, and central nervous systems adverse reactions, such as dizziness, headache and insomnia.^3^ Therefore, real-time detection of trace amounts of IL-6 and oseltamivir in body fluids with low cost would be highly relevant to mitigate COVID-19 propagation.^1^

Metal–organic frameworks (MOFs) are porous crystalline hybrid solids with a wide chemical and structural tunability.^4,5^ These materials have been considered so far for a broad range of potential applications including gas storage and separation, heterogeneous catalysis, biomedicine and sensing, including for COVID biomarkers.^6–8^ Among thousands of possible MOF compositions, yttrium based MOFs^9–11^ possess several attractive features such as high coordination numbers associated with a good chemical and thermal stability while their empty 5d orbitals are of interest from the view of electron transfer processes. Furthermore, due to faster mass diffusion, nanoparticles of MOFs, also denoted as nanoMOFs, have been shown to exhibit superior performances for adsorption, membrane design, and sensing.^10–13^ Moreover, defective nanoMOFs can exhibit extra active sites of interest to enhance host–guest interactions.^14–20^

A conventional strategy to detect a specific biomarker is to select an aptamer functionalized with a luminescent dye such as carboxyl fluorescein.^11–22^ To produce highly effective aptamer sensors, the secondary structure of nucleic acids in aptamers plays an essential role in their biological functions *in vivo*. In addition, using carboxyfluorescein (FAM)-labeled DNA aptamers with a stem loop structure has been considered an efficient way to shorten the distance between luminescent species, which may lead to a high luminescence resonance energy transfer (FRET) efficiency between the aptamer luminescent group and the second luminescent moieties such as the constitutive ligand from nanoMOFs.^20–22^ Therefore, designing a sensor based on the combination of a luminescent nanosized Y-MOF and DNA aptamer with a stem loop structure might lead to an unprecedented sensing platform for IL-6 and oseltamivir with concomitant high sensitivity and selectivity. Combining a DNA aptamer and MOF as a hybrid sensing platform is a well-established protocol.^23–25^ For example, Chen and co-workers have reported a MOF based fluorescence sensor for specific recognition of duplex DNA sequences.^26^ However, there are still challenges to address such as finding a stable sensing platform with good compatibility for the DNA and achieving a highly efficient FRET effect.^27^

**Scheme 1:**
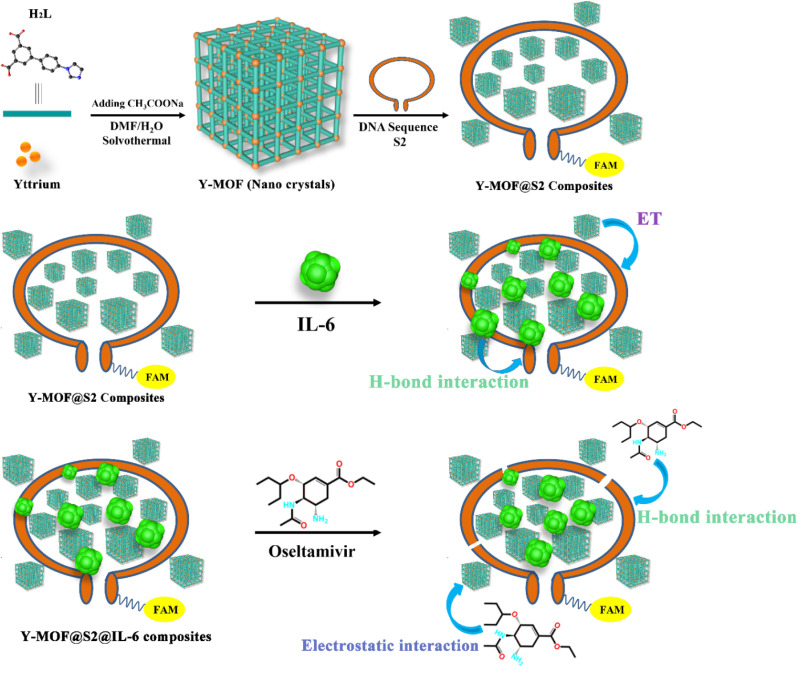
The proposed detection mechanism for IL-6 and oseltamivir based on a Y-MOF/DNA sequence luminescent sensing platform.

In this work, a new yttrium-based imidazole dicarboxylate MOF, namely Y-MOF [Y_2_(L)_3_(DMF)(H_2_O)_1.5_]_*n*_ (H_2_L = 5-(4-(imidazole-1-yl) phenyl)isophthalic acid) is reported. By tuning the synthesis parameters, this Y-MOF was prepared as nanoparticles possessing an increased concentration of open metal sites at their external surface (Scheme 1). A FAM labeled DNA sequence, named S1 was then selected due to its particular interactions with IL-6.^28^ To further enhance the electrostatic interactions between the DNA sequence and IL-6, extra adenine and thymine units were added to the head and tail parts of S1, to give another new DNA sequence, named S2 with a stem-loop structure. The combination of the nanosized Y-MOF and S2 led to the hybrid sensing platform Y-MOF@S2, which can efficiently detect IL-6 thanks to the modulation of its emission by energy transfer processes.

In addition, IL-6 was further combined with Y-MOF@S2 leading to the hybrid sensing platform Y-MOF@S2@IL-6, resulting in a specific recognition of oseltamivir among the potential COVID-19 drugs. The composite detection platform could finally be successfully used in human body fluids, with a linear detection range of IL-6 (5–15 pg mL^−1^) and oseltamivir (0.05–0.64 μM) that exceeds the maximum dangerous value for humans, making this Y-MOF/DNA sequence hybrid platform appealing for medical diagnostics.

## Results and discussion

### Synthesis and structure of Y-MOF

**Fig. 1:**
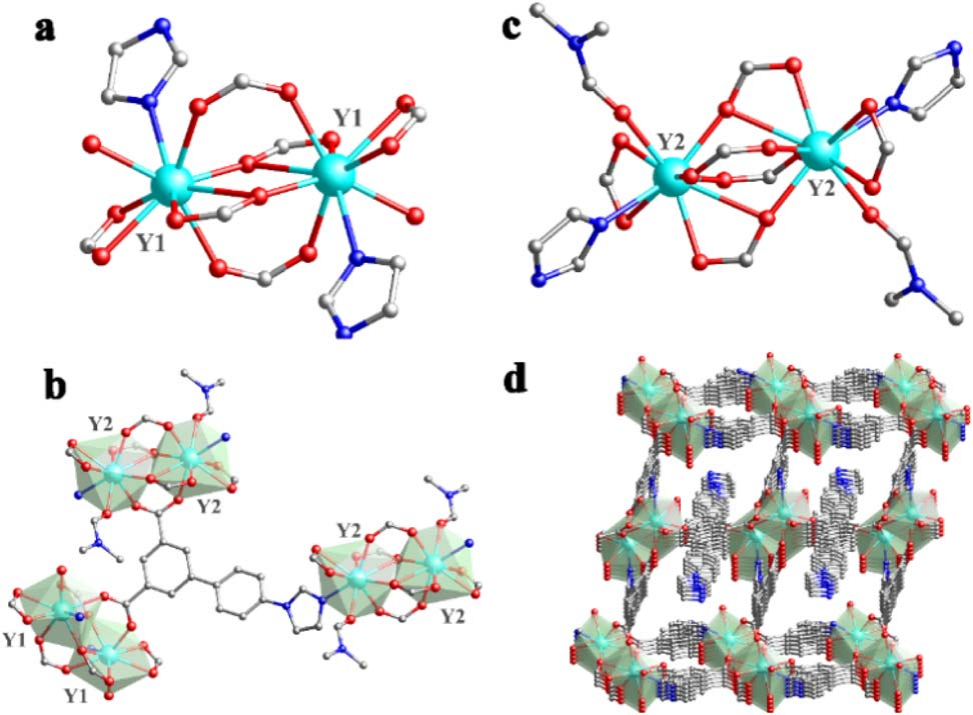
(a) Coordinating mode of the Y1 site; (b) coordinating mode of the Y2 site; (c) fundamental structural unit of Y-MOF; (d) three-dimensional oxocluster-based structure of Y-MOF.

The Y-MOF was synthesized under solvothermal conditions in DMF (dimethylformamide) with a H_2_L ligand (H_2_L = 5-(4-(imidazole-1-yl) phenyl)isophthalic acid) and YCl_3_ at 120 °C over 3 days (see details in the ESI†) leading to single crystals of suitable dimensions for structure determination. Single-crystal X-ray diffraction analysis evidenced that Y-MOF [Y_2_(L)_3_(DMF)(H_2_O)_1.5_]_*n*_ crystallizes in a triclinic system, with a *P*1̄ (no. 2) space group (Table S1†). As depicted in Fig. 1a, the asymmetric unit of Y-MOF consists of two independent Y centers (Y1 and Y2), three entirely deprotonated L^2−^, one coordinated DMF molecule and one coordinated H_2_O molecule.

Each central Y^III^ ion (Y1) is nine-coordinated by seven oxygen atoms (O1, O1A, O2, O3A, O4A, O5, and O6A), one nitrogen atom N2A from L^2−^ and one oxygen atom (O13) from one coordinated H_2_O (Fig. 1b). The other Y^III^ ion (Y2) is also nine-coordinated by seven oxygen atoms (O7, O10, O10A, O8, O9, O11, and O12), one nitrogen atom N4 from L^2−^ and one oxygen atom (O15) from coordinated DMF (Fig. 1c). As the secondary building unit (SBU) of Y-MOF, two Y1 atoms and two Y2 atoms form two eight-membered rings (Y_2_O_4_C_2_) respectively through the carbon and oxygen atoms of the ligand, in which four-membered Y_2_O_4_ oxoclusters are nested internally to create a binuclear cage-like structure. The Y–O bond lengths vary from 2.282 to 2.779 Å, and O–Y–O bond angles range from 49.49° to 153.55° (Tables S2 and S3†). On the other side, by adopting multi-dentate bridging modes, each L^2−^ ligand links three neighboring binuclear cage-like structures, further constructing the tridimensional framework (Fig. 1d). In addition, C–H⋯O hydrogen bonds for Y-MOF (Table S4†) may contribute to the good chemical stability of the framework. The PLATON program demonstrates that the overall potential solvent volume is 89.3 Å^3^ per unit-cell, which corresponds to 3.5% of the unit-cell volume (2580 Å^3^).

### Optimization of the amount of open metal sites in Y-MOF (1)–(3)

Herein, to increase the amount of open metal sites on the Y-MOF particle external surface, Y-MOF (1)–(3) were synthesized under solvothermal conditions by introducing sodium acetate (CH_3_COONa) as a modulator. Concentrations of sodium acetate of 5 mM and 10 mM in the reaction system (1 mL, water as solvent) were considered to prepare compounds (2) and (3), as sodium acetate can create defects in the Y-MOF because it competes with the “–COOH” group in H_2_L during the synthesis to coordinate with Y^3+^ ions.^29^ PXRD, TGA and variable temperature PXRD all confirmed that samples (1)–(3) exhibit the same crystal structure (see Fig. S2 and S3†) and also revealed that Y-MOF is stable under an oxygen atmosphere until 400 °C. SEM analysis showed that the average particle size is 106 (27) nm for (1), 109 (24) nm for (2) and 118 (22) nm for (3), in good agreement with DLS measurements (Fig. S1 and Table S5†). The amount of acetates in the final products was assessed by liquid phase ^1^H-NMR carried out on the MOF digested solution, indicating a H_2_L ligand to CH_3_COO^−^ ratio of 30 for (2) against 12 for (3) (Fig. S4†), in agreement with ligand defects leading to an increasing amount of open metal sites in Y-MOF (3).^10^

### Y-MOF@S2 hybrid sensing platform for the detection of IL-6

To achieve a highly specific detection of IL-6, a specific FAM labeled IL-6 aptamer namely S1, was selected.^22^ Extra adenine and thymine units were added to the head and end parts of S1, resulting in a new sequence, named S2 to further enhance the interactions between the aptamer and IL-6. The zeta potentials of S1 (1 μM), S2 (1 μM), and IL-6 (0.48 μM) were −4.8 mV, 2.3 mV and −12.4 mV in water solution (Fig. S5†), indicating that S2 can combine with IL-6 through electrostatic interactions. To confirm this, a nanoMOF suspension (0.1 g L^−1^, 900 μL) was mixed with the DNA sequence S2 (1 μM, 100 μL) for 30 minutes to anchor S2 at the surface of the Y-MOF and form the hybrid sensing platform Y-MOF@S2. Upon excitation at 380 nm, pure Y-MOF emitted at 430 nm while the FAM labeled DNA sequence S2 emitted at 520 nm (Fig. S6 and S7†), confirming their compatibility to build a hybrid sensing platform with high FRET efficiency. The luminescence spectrum of Y-MOF@S2 indeed presented two emission peaks under excitation at 380 nm: one located at 433 nm issued from the Y-MOF constitutive ligand, and a second located at 514 nm attributed to the FAM group on S2. In order to get an efficient sensor, the relative concentrations of MOF and S2 were optimized in order to get a luminescent signal of similar intensity for both components (Fig. S6†). The emission intensities of Y-MOF, S2 and Y-MOF@S2 were then compared. When Y-MOF and S2 were mixed to prepare Y-MOF@S2, the relative emission intensity of Y-MOF in Y-MOF@S2 decreased, while the relative emission intensity of FAM labeled S2 increased, suggesting the presence of a Förster resonance energy transfer (FRET) between the H_2_L ligand in Y-MOF and S2 (H_2_L acts as an energy donor and S2 acts as the energy acceptor (Fig. S7a†)). We further excluded the possibility of FAM in S2 acting as an energy acceptor (Fig. S7b†). Indeed, after pure FAM was added into the nanoMOF solution, the emission intensity of Y-MOF did not change. Furthermore, upon excitation at 260 nm, the lifetime associated with the emission at 370 nm decreased from 1.57 ns to 1.46 ns after adding S2 gradually into the Y-MOF suspension (Table S6 and Fig. S8†), which further confirmed the existence of the FRET process between the Y-MOF ligand and S2.

**Fig. 2:**
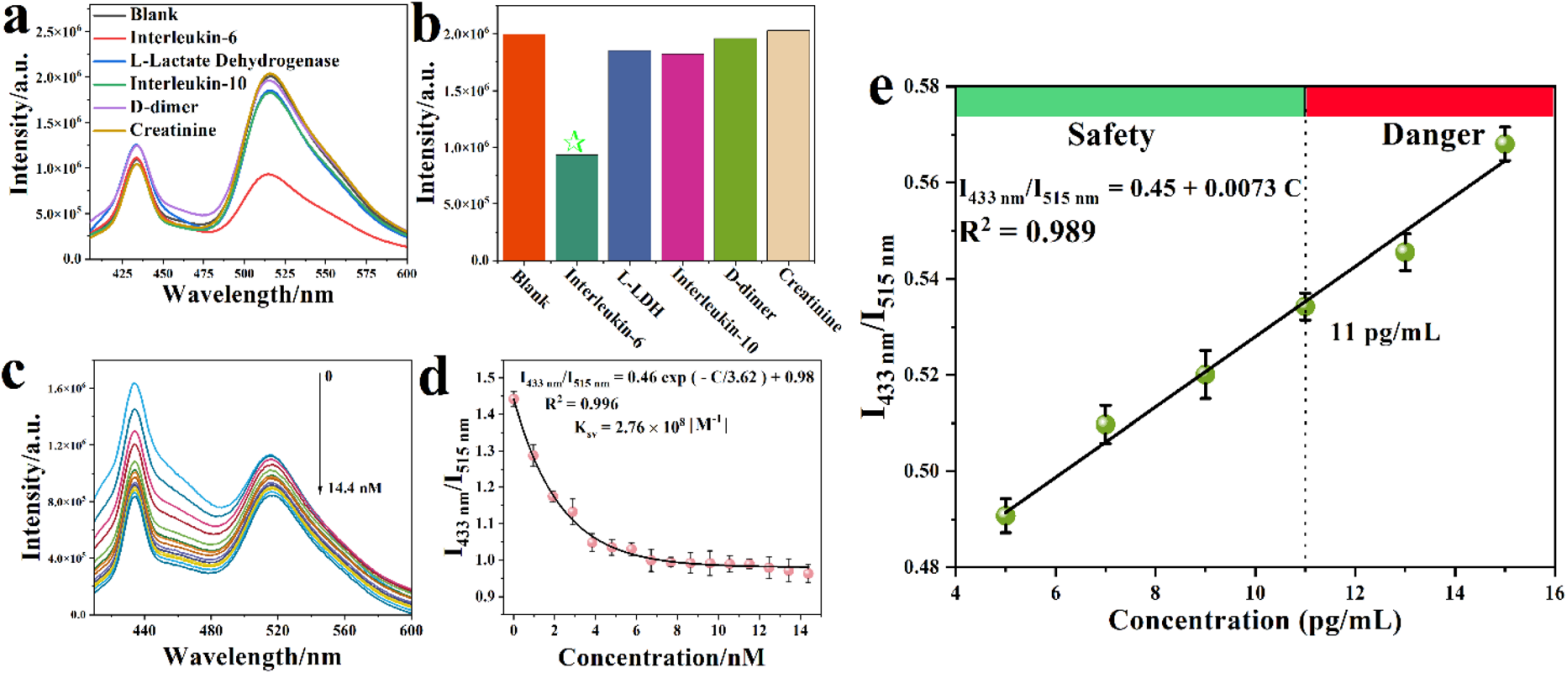
(a) Luminescence spectra of Y-MOF(3)@S2 after adding different COVID-19 biomarkers (1 μM, water as solvent), excitation at 380 nm; (b) luminescence intensity at 515 nm of Y-MOF(3)@S2 after adding different biomarkers (1 μM, water as solvent); (c) luminescence spectra of Y-MOF(3)@S2 after adding different concentrations of IL-6 in human blood solution (5 μL water as solvent) (excitation at 380 nm); (d) non-linear relationship between the concentration of IL-6 and the luminescence intensity ratio of Y-MOF(3)@S2 in human blood solution (5 μL water as solvent); (e) the linear relationship between the concentration of IL-6 and the luminescence intensity ratio of Y-MOF(3)@S2 in human body fluids.

To evaluate the potential of Y-MOF@S2 as a ratiometric luminescent sensor for COVID-19 biomarkers, we selected a d-dimer, L-lactate dehydrogenase (L-LDH), IL-10, creatinine and IL-6 as biomarker candidates, as they have all been proven to be relevant to COVID-19. Indeed, elevated levels of these biomarkers have been identified in non-survivors compared with survivors throughout the clinical course, and their amounts increased with illness deterioration.^1^ Thus, IL-6, L-LDH, IL-10, d-dimer and creatinine were added into Y-MOF@S2 suspensions at the same concentration (1 μM). Only IL-6 induced an obvious quenching effect on Y-MOF@S2 at 514 nm (Fig. 2a and b), in agreement with the ability of the S2 aptamer to selectively interact with IL-6. The lack of strong interactions with the other biomarkers is of strong practical interest for the selective detection of increasing concentrations of IL-6 under real conditions (*e.g.* human blood). With Y-MOF(3), the relationship between the luminescence ratio at 433 nm and 515 nm and the concentration of IL-6 followed a non-linear Stern–Volmer (S–V) behavior with different quenching rates within the concentration range of 0–14.4 nmol L^−1^ (Fig. 2c). A *K*_SV_ value of 2.76 × 10^8^ M^−1^ was obtained based on the fitting results using the empirical equation *I*_1_/*I*_2_ = *a* exp(*K*_SV_[C]) + *b* for IL-6 (Fig. 2d).^16^ The limit of detection (LOD) for IL-6 was found to be 70 pM according to the equation: LOD = 3*S*_0_/*S* (where *S*_0_ is the standard deviation for the blank and S is the slope of the calibration curve). The recovery tests were carried out in human blood and ranged from 97.8% to 103.1%, with a relative standard deviation (RSD) below 1.87% (Table S7†), which confirmed the good reproducibility of this detection method. In addition, we also compared the sensitivity of Y-MOFs (1–3) towards the IL-6 detection and found a lower detection sensitivity for Y-MOF(2)@S2 and Y-MOF(1)@S2 (Fig. S9 and S10†). This could be due to the decreased number of available open metal sites on the external surface of the nanoMOF that may weaken the electronic communication between the Y-MOF and FAM from S2. The evolution of the emission spectra of Y-MOF(3)@S1, Y-MOF@S2,Y-MOF@S3 (S3 is a mutated aptamer for S2), pure S2 and pure Y-MOF in the presence of IL-6 was also compared (Fig. S11–S13†). Interestingly, Y-MOF(3)@S2 exhibited, by far, the strongest quenching effect, highlighting the high affinity between S2 and IL-6 and therefore, the relevance of our new sensing platform design.

In order to further assess the potential of this hybrid sensing platform for IL-6 detection in COVID-19 patients, the behavior of Y-MOF(3)@S2 in the concentration range where IL-6 becomes dangerous in simulated body fluid, which contains other COVID-19 biomarkers as we mentioned before and which are controlled under normal concentrations in the human body (the maximum content of IL-6 is 11 pg mL^−1^ in a healthy body), was analysed.^2,24–28^ Noteworthily, as shown in Fig. 2e, a linear relationship between the luminescence intensity ratio (*I*_434nm_/*I*_515nm_) and IL-6 concentration was obtained.

### Sensing mechanism of IL-6

**Fig. 3:**
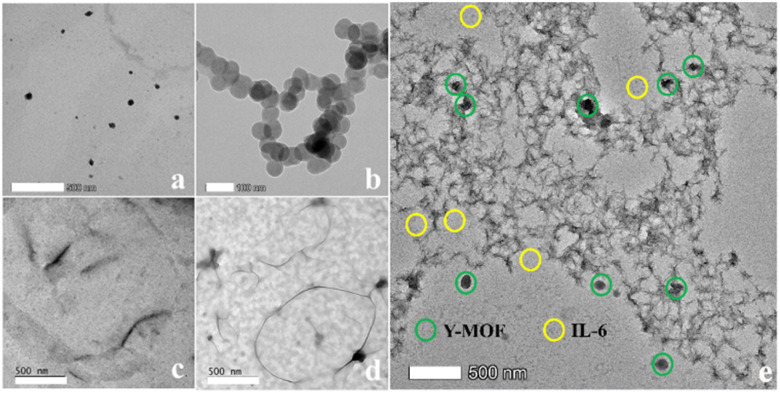
TEM images of (a) Y-MOF(3); (b) aggregates of IL-6; (c) DNA sequence S1; (d) DNA sequence S2; (e) a mixture of Y-MOF(3), S2 and IL-6.

The mechanism behind the selective IL-6 detection was analyzed based on the following advanced characterization studies: (1) powder XRD showed that the MOF structural integrity was maintained after being immersed in IL-6 and S2 solutions, thus confirming that the quenching effect is not due to a collapse of the framework (Fig. S2†). (2) According to TEM images, IL-6 and nanoMOFs are located around S2 DNA loops (Fig. 3). The DLS test also revealed that the size of the Y-MOF@S2 detection system increased to 5 μm after adding IL-6. (3) Circular dichroism spectra revealed that DNA sequence S1 is associated with a chiral signal, while S2 is achiral due to its stem-loop structure. Therefore, we used S1 instead of S2 to explore the nature of the interactions between the Y-MOF, IL-6 and DNA sequence using circular dichroism. After introducing IL-6 and Y-MOF into S1 solution (Fig. S14†), the dichroic signal nearly disappeared indicating that IL-6 and Y-MOF combine with S1 and modify the secondary structure of the chiral aptamer. (4) Energy transfer between the donor, *i.e.* the ligand H_2_L in Y-MOF@S2 and the acceptor IL-6, was confirmed by the luminescence lifetime measurements. Under excitation at 260 nm, the lifetime of the ligand, located at 370 nm, decreased from 1.43 ns to 1.38 ns after adding IL-6, in agreement with the FRET process (Tables S8 and S11 and Fig. S15†).

**Fig. 4:**
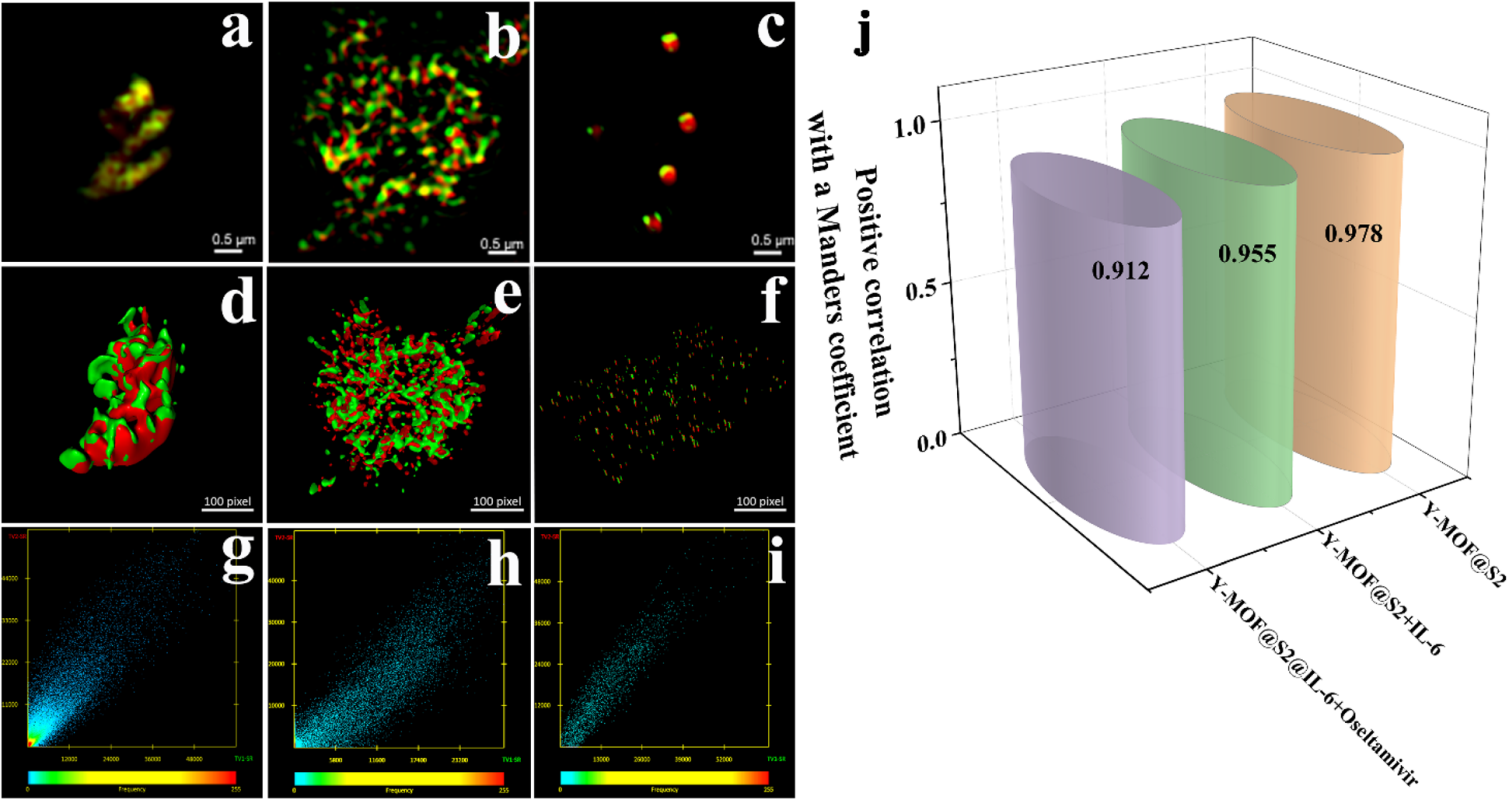
Overlay colocalization of (a) Y-MOF@S2; (b) Y-MOF@S2 after adding IL-6; (c) Y-MOF@S2@IL-6 after adding oseltamivir by SIM respectively; clockwise 3D rendering of (d) Y-MOF@S2; (e) Y-MOF@S2 after adding IL-6; (f) Y-MOF@S2@IL-6 after adding oseltamivir, respectively; fluorescence intensity correlation of (g) Y-MOF@S2; (h) Y-MOF@S2 after adding IL-6; (i) Y-MOF@S2@IL-6 after adding oseltamivir between the red signal corresponding to Y-MOF and the green signal corresponding to S2; (j) positive correlation with a Manders coefficient of Y-MOF@S2 under different conditions.

Fluorescence microscopy has become a valuable tool in cell biology research to analyze fluorophore-tagged proteins, DNA, RNA and their interactions. The resolution obtained by *epi*-fluorescence microscopy is limited by Abbe's diffraction limit, defined as the minimum distance between two closely localized structures that can be distinguished from each other.^30^ The achieved resolution in biological specimens is restrained to ∼200–250 nm in the lateral plane and ∼500–700 nm in the axial dimension.^31^ This limit can be overcome by fluorescence nanoscopy, also referred to as super-resolution microscopy. This technique is based on structuring illumination light obtained using a wide-field configuration resulting in three-dimensional (3D) structured-illumination microscopy (SIM). In this study, we used SIM to estimate the distance between Y-MOF and S2. From the SIM images (Fig. S16†), nanoMOFs and S2 with a stem loop structure could be observed. We used the Manders coefficient^31–34^ to evaluate the overlap degree between Y-MOF and S2 and found a positive correlation with a Manders coefficient decreasing from 0.978 to 0.955 after adding IL-6, which indicated that IL-6 interrupts the FRET process between Y-MOF and S2 (Fig. 4).

**Fig. 5:**
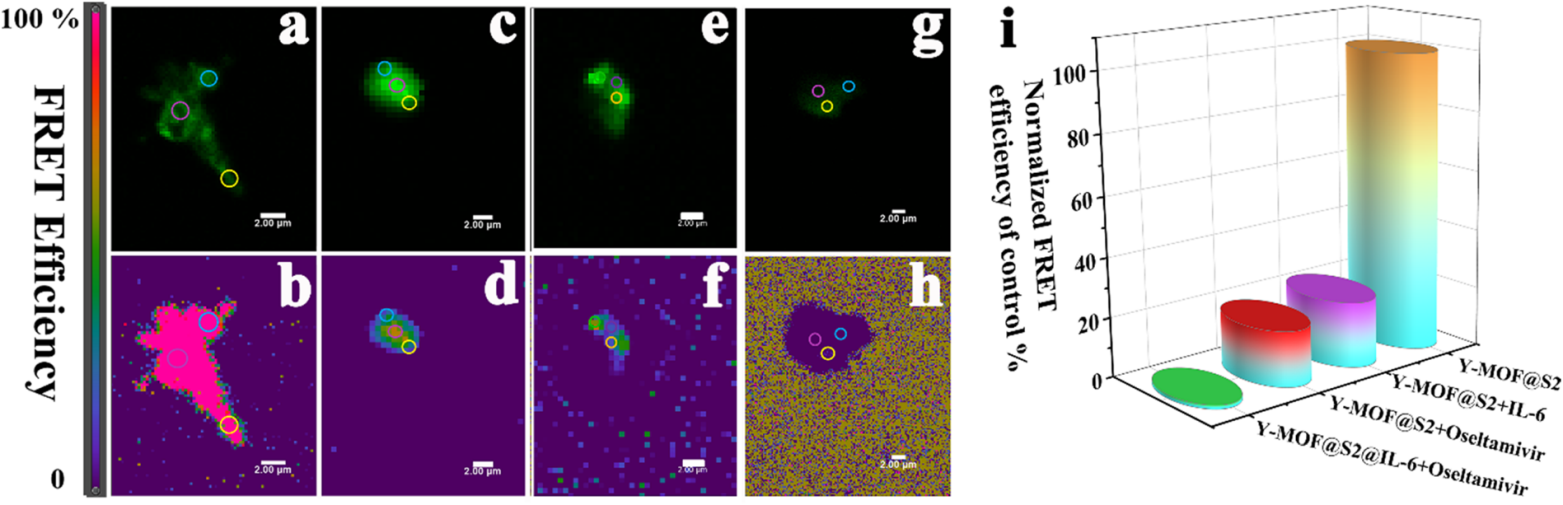
Confocal microscope images of Y-MOF@S2 under different conditions: (a) Y-MOF@S2 in the FRET channel; (b) FRET efficiency of Y-MOF@S2; (c) Y-MOF@S2 after adding IL-6 in the FRET channel; (d) FRET efficiency of Y-MOF@S2 after adding IL-6; (e) Y-MOF@S2 after adding oseltamivir in the FRET channel; (f) FRET efficiency of Y-MOF@S2 after adding oseltamivir; (g) Y-MOF@S2@IL-6 after adding oseltamivir in the FRET channel; (h) FRET efficiency of Y-MOF@S2@IL-6 after adding oseltamivir; (i) normalized FRET efficiency of Y-MOF@S2 under different conditions.

To gain a deeper understanding of the energy transfer mechanism between Y-MOF and S2, the FRET sensitive emission (FRET SE) method was applied using confocal microscopy and structured illumination microscopy (SIM). Sensitized emission (SE)-FRET is a fluorescence intensity-based method that uses changes in fluorophore spectra to measure FRET.^35,36^ In this work, we have applied this method to evaluate FRET efficiency of Y-MOF@S2 to gain understanding of the quenching mechanism. After adding IL-6, the FRET efficiency in the Y-MOF@S2 platform decreased from 100% to 15.1% (Fig. 5). On the other hand, luminescence emission of the FAM-labeled DNA sequence was quenched by IL-6, due to hydrogen bonds between IL-6 and S2. This was confirmed by FT-IR spectroscopy whereas, upon addition of IL-6 into Y-MOF(3)@S2, the “–OH” stretching bands centered at 3650 cm^−1^ from the nanoMOF open metal sites were gradually blue-shifted and became more intense, associated with longer O–H distances (Fig. S17†).^37–39^ As a result, the sensing mechanism for the efficient detection of IL-6 may be ascribed to the interruption of the FRET process between the nanoMOF and S2 due to the appearance of hydrogen-bond interactions at the nanoMOF outer surface with S2 and IL-6, leading to quenching effects during the detection process of IL-6.

### Highly efficient detection of oseltamivir based on Y-MOF/DNA sequence S2/IL-6

**Fig. 6:**
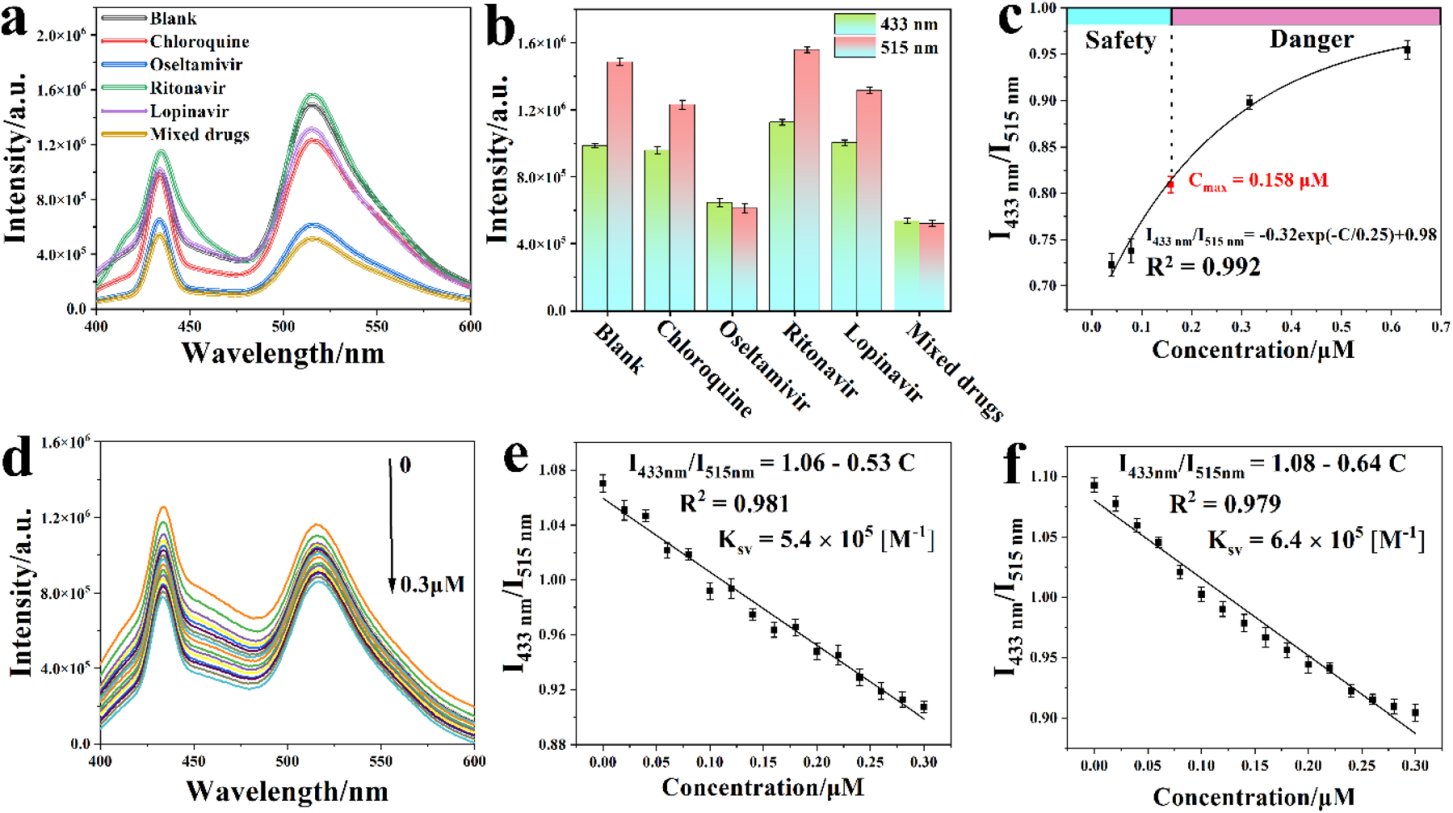
(a) Luminescence spectra of Y-MOF(3)@S2@IL-6 after adding different COVID-19 drugs (1 μM); the mixed drug solution (DMSO as solvent) is a combination of chloroquine, oseltamivir, ritonavir, and lopinavir, (excitation at 380 nm); (b) luminescence intensity of Y-MOF(3)@S2@IL-6 at 433 nm and 515 nm after adding different COVID-19 drugs (1 μM); (c) the non-linear relationship between the concentration of oseltamivir and the luminescence intensity ratio of Y-MOF(3)@S2@IL-6 in human body fluids; (d) luminescence spectra of Y-MOF(3)@S2@IL-6 in human blood solution (5 μL water as solvent) after adding different concentrations of oseltamivir (the detection concentration range is from 0 to 0.30 μM, excitation at 380 nm); (e) linear relationship between the luminescence intensity ratio of Y-MOF(3)@S2@IL-6 and concentration of oseltamivir in human blood solution (5 μL, water as solvent); (f) linear relationship between the luminescence intensity ratio of Y-MOF(3)@S2@IL-6 and concentration of oseltamivir in the mixed drug solution; the mixed drug solution (DMSO as solvent) is a combination of chloroquine, oseltamivir, ritonavir and lopinavir (excitation at 380 nm).

The potential detection ability of Y-MOF(3)@S2@IL-6 towards various COVID-19 drugs, including chloroquine, oseltamivir and ritonavir, was assessed. Emission spectra first evidenced that the luminescence intensity of Y-MOF(3)@S2@IL-6 at 430 and 514 nm was quenched after adding a 1 μM oseltamivir solution (Fig. 6a and b). In contrast, the other COVID-19 drugs did not influence the luminescence intensity of Y-MOF(3)@S2@IL-6 in the same way, confirming the excellent selectivity of our system towards oseltamivir. The quenching efficiency of Y-MOF(3)@S2@IL-6 was then evaluated according to the equation

1 *I*_433nm_/*I*_515nm_ = *C* + *K*_sv_·[A],

which gives a *K*_sv_ value of 5.4 × 10^5^ M^−1^ for Y-MOF(3)@S2@IL-6 in a low concentration range (0–0.3 μM) with an excellent linear fit (*R*^2^ = 0.988) (Fig. 6e). Noteworthily, oseltamivir could be detected using Y-MOF(3)@S2@IL-6 with high efficiency even in mixed potential Covid-19 drug solution (Fig. 6f). To explore the practical application of oseltamivir detection, the sensing potential in the concentration range where oseltamivir becomes dangerous in the human body (indicated by the maximum plasma concentration *C*_max_) was also investigated. As shown in Fig. 6c, a non-linear relationship between the luminescence intensity ratio (*I*_433nm_/*I*_515nm_) and oseltamivir concentration was obtained until *C*_max_, which suggests that a combination of dynamic and static quenching effects occurs in high concentration oseltamivir detection (Fig. 6d and e). Our system was also compared with the bare Y-MOF and S2 to detect oseltamivir and although a quenching effect was observed in both cases, it was associated with a lower sensitivity and stability, further justifying the interest of our sensing platform (Fig. S18–S21 and Table S9†).

### Microscopic insight into the interaction between oseltamivir and Y-MOF

Density functional theory (DFT) calculations were further performed to gain microscopic insight into the MOF/oseltamivir interactions. Since the kinetic diameter of the oseltamivir molecule exceeds by far the pore size of this non porous Y-MOF, this guest is expected to interact only with the external surface of the MOF.

**Fig. 7:**
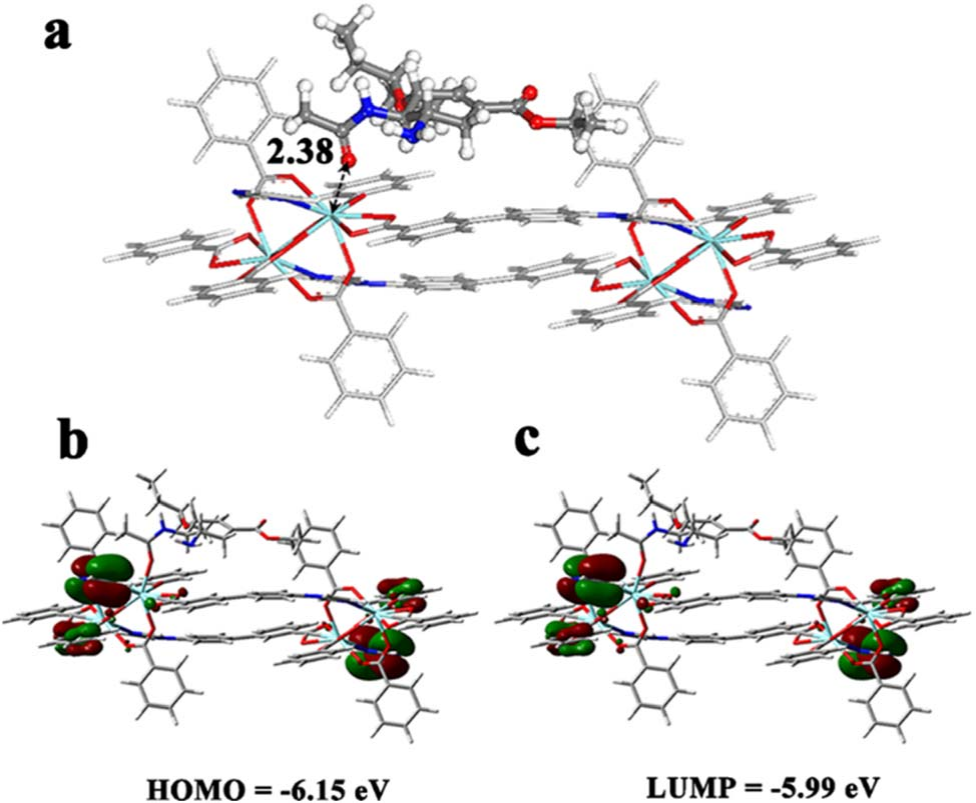
(a) DFT optimized geometry of the oseltamivir molecule (ball and stick) interacting with the Y-MOF representative cluster (stick). The characteristic interacting distances are reported in Å. Color codes for all atoms: hydrogen (white), carbon (light grey), nitrogen (blue), oxygen (red) and yttrium (cyan); (b) the frontier molecular orbital (HOMO and LUMP) diagrams for the oseltamivir/MOF cluster. In the MOs, red and green colors indicate negative and positive charges respectively.

Indeed, a cluster model containing Y-open metal sites potentially present at the nanoMOF surface and likely exposed to the oseltamivir molecules was then cleaved from the crystal structure (see Fig. S23†) and loaded with 1 oseltamivir molecule. The most energetically favorable conformation of oseltamivir was taken from a previous study.^40^ Two distinct binding modes for oseltamivir were examined *i.e.* (i) a single adduct towards the Y-metal site *via* its carbonyl function and (ii) chelating species implying its carbonyl and amine functions with the consideration of multiple starting configurations. Fig. 7a reveals that oseltamivir adopts a preferential orientation in such a way as to interact with Y-metal sites *via* its carbonyl group, associated with a relatively short O_(CO)_–Y distance of 2.38 Å. This single adduct is likely due to the steric hindrance around the Y-metal sites that prevents the formation of a chelation geometry reported previously for the exposed metal in doped fullerene. The calculated interaction energy for the resulting oseltamivir/Y-MOF complex was found to be −76.6 kJ mol^−1^, in line with the rather high affinity observed experimentally. Frontier molecular orbitals (MOs) were further analyzed to evaluate the stability of the oseltamivir/Y-MOF complex. Fig. 7b illustrates the charge density localization and energy transfer within the MOs of the complex. Only a tiny change was observed in the energies of the HOMO and LUMO (−6.15 eV and −5.99 eV respectively) and the total charge density remains localized over the organic linkers.

### Sensing mechanism of oseltamivir

To discuss the reason why oseltamivir exhibits a high quenching effect towards Y-MOF(3)@S2@IL-6, one shall consider the following points. First, the DLS test also reveals that the size of the Y-MOF@S2@IL-6 detection system reaches 3 μm after adding oseltamivir. During the oseltamivir detection process, oseltamivir can quench the luminescent emission of pure Y-MOF (Fig. S19†). After adding oseltamivir into Y-MOF(3)@S2@IL-6, the lifetimes at 370 nm (Y-MOF emission position) and at 514 nm (S2 emission position) did not vary (Fig. S22†), which is in line with the physisorption-based interactions between open Y-metal sites and the carbonyl group of oseltamivir revealed by DFT calculations (Fig. 7). In addition, after introducing oseltamivir into IL-6 solution, the chiral signal of IL-6 in the CD spectrum decreased, which revealed that oseltamivir may combine with IL-6 and induce a change in its chiral construction that can drastically affect the steric configuration of IL-6, thus explaining why Y-MOF(3)@S2@IL-6 exhibits a more sensitive detection for oseltamivir than Y-MOF(3)@S2. More importantly, structured-illumination microscopy evidenced that the addition of oseltamivir into Y-MOF@S2@IL-6 led to the disconnection of the stem loop structure thus associated with a disappearance of the energy transfer ability of Y-MOF@S2 leading to a strong quenching effect towards Y-MOF@S2@IL-6, as the overlap degree between Y-MOF and S2 sharply decreased (Fig. 4). Furthermore, oseltamivir also influenced the energy transfer between Y-MOF and S2, as observed by confocal microscopy (Fig. 5). Finally, after adding oseltamivir, FRET efficiency almost disappeared (relative to FRET efficiency of Y-MOF@S2). IL-6 also played an important role during the detection process, as when oseltamivir interacts with IL-6, amino groups from oseltamivir are expected to interact with the carboxylates from IL-6 and therefore interrupt the hydrogen bonds from the disulfide bond and consequently interrupt the FRET process between Y-MOF and the S2 sequence (Tables S10, S12 and Fig. S19b†).

## Conclusions

In this work, we report for the first time the selective detection of COVID-19 biomarker IL-6 and potential curing drug oseltamivir based on new ratiometric luminescent defect engineered Y-MOF nanoparticles coated with a flexible DNA based stem loop. Due to hydrogen bonds and π⋯π stacking interactions, IL-6 and oseltamivir can attract and disconnect the stem loop structure of S2, which leads to a strong quenching effect on the Y-MOF@S2 emission. Y-MOF@S2 and Y-MOF@S2@IL-6 showed high sensitivity towards IL-6 and oseltamivir at a low level of detection and both sensing platforms can detect these analytes under real conditions. The nature of the MOF/interactions was explored computationally and the detection mechanism of IL-6 and oseltamivir was fully characterized by confocal microscopy and luminescence lifetime tests to shed light on the selective FRET detection of this robust and selective new hybrid biosensor. This work paves the way for the future design of new generations of MOF-DNA based sensing devices of COVID-19 biomarkers and potential drugs.

## Data availability

Experimental data are available upon request.

## Author contributions

The original idea was conceived and experimental data analysis was performed by Xinrui Wang under the supervision of Antoine Tissot, Christian Serre, Bin Ding and Gilles Clavier. Structure characterization and TEM tests were performed by Bin Ding, and luminescence decays were performed by Gilles Clavier. The original manuscript was first drafted by Xinrui Wang and revised by the main authors. LSCM and SIM images were obtained by Nannan Xiao and Yan Zhang. DFT calculations were carried out by Kamal Batra under the supervision of Guillaume Maurin. All authors have participated in the modification of the manuscript and given approval to the final manuscript.

## Conflicts of interest

There are no conflicts of interest to declare.

## Supplementary Material

SC-014-D3SC00106G-s001

SC-014-D3SC00106G-s002
