# Supplementary material for: A MOF/DNA luminescent sensing platform for detection of potential COVID-19 biomarkers and drugs
Source: Chem Sci. 2023 Apr 19;14(20):5386–95. doi: 10.1039/d3sc00106g (PMC10207894; doi:10.1039/d3sc00106g)
Supplement: SC-014-D3SC00106G-s001 [file SC-014-D3SC00106G-s001.pdf]

Electronic Supplementary Material (ESI) for Chemical Science.
This journal is © The Royal Society of Chemistry 2023

Supporting information

MOF/DNA luminescent sensing platform for detection of COVID-19 potential biomarker and drug

1. General methods

The H_2L Ligand was purchased from JiNan Henghua (Jinan, China). DNA sequences were purchased from AZENTA company. Human blood was collected from HuanHu hospital (China). All the other reagents without further purification and were commercially purchased and utilized. C, H, and N microanalyses were carried out with a Perkin-Elmer 240 elemental analyzer. Powder X-Ray Diffraction (PXRD) was characterized by a high-throughput Bruker D8 Advance diffractometer working on transmission mode and equipped with a focusing Göbel mirror producing CuKα radiation (λ = 1.5418 Å) and a LynxEye detector, utilizing Cu-Kα radiation. FT-IR spectra (4000- 500 cm^{-1}) were recorded by utilizing a Nicolet iS5 FTIR ThermoFisher spectrometer. Ultrasonic preparation was carried by a Branson Ultrasonic bath (sonifier sound enclosure Instrument, USA). Agilent technologies (USA) cary 300 spectrophotometer was utilized to recording ultraviolet-visible (UV-vis) adsorption spectra. The photo-luminescent spectrum and solid fluorescent spectrum were performed by Fluorolog-3, Horiba Jobin Yvon (USA).

Lifetime experiments were done using 10 mm path length quartz cuvettes: The ns fluorescence decay curves were obtained by the time-correlated single-photon counting (TCSPC) method. The setup is composed with a titanium sapphire Ti:Sa oscillator (Spectra Physics, Maï Taï HP) emitting pulses of 100 fs duration at 690nm, 80MHz frequency. The laser pulses then pass through a pulse picker which implements acousto-optic modulator to pick up specific pulse to reduce the repetition rate at 4MHz (GWU Lasertechnik, UHG-23-PSK). Then the beam, after adjusting the excitation power and the polarization with respectively an intensity attenuator filter wheel and a Fresnel rotator, passes through the sample solution. Fluorescence photons were detected at 90° through a long pass filter (Schott, RG9), a monochromator (CVI Laser Corporation, Digikröm CM110) and a polarizer by means of a micro channel plate photomultiplier (Hamamatsu,

MCP-PMT R3809U-50), connected to a TCSPC module (Becker & Hickl, SPC-630). Time-correlated fluorescence decay data is finally processed and analysed with the help of a software which implements the non-linear square method (Globals, Laboratory for Fluorescence Dynamics at the University of Illinois at Urbana-Champaign). DLS test and Zeta potential measurements were performed on Nano-ZS, Worcestershire, (Malvern, UK) at a typical concentration of 0.01g/L. MilliQ water was obtained from Millipore system. SEM images were recorded with FEI Magellan 400 scanning electron microscope. TGA data were collected on Mettler Toledo TGA/DSC 2, STAR System apparatus with a heating rate of 5 °C/min under the oxygen flow. Mettler Toledo FiveEasy™ Plus pH / mV bench meter. Infrared spectra were measured with a Nicolet iS5 FTIR ThermoFisher spectrometer. ICP analyst was carried by Agilent 7700 Series ICP-MS, USA.

2. X-ray Crystallography.

Diffraction intensities for Y-MOF were performed on a Bruker SMART 1000 CCD diffractometer utilizing graphite-monochromated Cu-Kα radiation (λ = 0.71073 Å). Diffraction intensities for Y-MOF were performed utilizing graphite-monochromated Mo-Kα radiation. Lorentz polarization, ω-φ scanning method and absorption corrections were utilized. The structures were analyzed by direct methods and refined with the full-matrix least-squares method utilizing the SHELXS-97 and SHELXL-97 programs. Anisotropic thermal parameters were assigned to all non-hydrogen atoms. Organic hydrogen atoms were defined geometrically. Analytical expressions of neutral-atom scattering factors were utilized, and anomalous dispersion corrections were incorporated. Crystallographic data and refinement details for 1 were summarized in Table S1, selected bond lengths and angles were summarized in Table S2-S5 and selected hydrogen bonds lengths [Å] and angles [°] were listed in Tables S6-S7 which calculated with the program PLATON. CCDC-2151322 (1), contain the crystallographic data for this work. These data can be obtained free of charge from Cambridge Crystallographic Data Centre via www.ccdc.cam.ac.uk/data_request/cif.

3. Fluorescence images

The high resolution fluorescent images were carried by the super-resolution microscope

Elyra 7 with SIM mode and FRET sensitized emission (FRET SE) images of Y-MOF@S2 under different conditions calculated by Leica SP8 Confocal Microscope.

The shooting conditions of the high resolution fluorescent images are carried in the same condition: The objective name is Plan-Apochromat 63X/1.4 Oil DIC M27, and we apply two laser device the excitation position are 405 nm (intensity: 60%) and 488 nm (intensity: 10%). We select two channels to collect fluorescent signals in the images: one channel for observing Y-MOF is from 405 nm to 440 nm which labeled in red, when excited at 405 nm; the other one for observing S2 is from 488 nm to 515 nm which labeled in green (excited at 488nm).

In the FRET process, an excited fluorophore (donor) transfers its excited state energy to a light absorbing molecule, i.e. the so-called acceptor. Sensitized Emission is a powerful tool to evaluate the FRET efficiency particularly for living cells as well as fixed samples. Due to the necessary overlap between donor emission spectrum and acceptor absorption spectrum, each FRET measurement is done by sequential acquisition of donor channels in confocal microscope. Donor and acceptor pairs can be used to generate data combined to control experiments giving background corrections that allow determining the degree of FRET.

The shooting conditions of FRET SE images are possessed in the same condition: The objective name is HC PL APO CS 40x/1.30 OIL, We select two channels to collect fluorescent signals in the images: one channel for observing Y-MOF (donor channel) is from 420 nm-490 nm, when excited at 405 nm; the other one for observing S2 (acceptor channel) is from 510 nm -580 nm which labeled in green (excited at 405 nm). The intensity of laser (405 nm) is 10%. FRET efficiency can be calculated by the following equation: $E_A = \frac{B - A\times\beta - C\times\gamma}{C}$, E_A is the apparent FRET efficiency, A, B, C correspond to the intensities of the three signals (donor, FRET, acceptor) and β, γ are the calibration factors generated by acceptor only and donor only.

2. Computational Details

A dimethylformamide solvent-free version of Y-MOF was first obtained by removing the dimethylformamide molecule coordinated to the Y-metal sites from the experimental crystal structure. The cluster model containing Y-open metal sites potentially present at the MOF surface cleaved from this 3D structure is represented in **Figure S23**. The cluster was loaded with

one oseltamivir molecule and further geometry-optimized at the Density Functional Theory (DFT) level. To ensure an efficient sampling of the electronic potential energy surface, multiple initial configurations were examined. These DFT calculations were performed using Gaussian 09, [2] employing B3LYP functional [3, 4] along with Grimme's D3-BJ correction. [5, 6] The 6-31G(d) basis set [7] was employed for H, C, N, and O atoms, while a 28-electron quasi relativistic effective core potentials and the segmented basis sets MWB28 were used for Y^{3+} metal ions. [8, 9] In order to retain the local geometry of the MOF structure, it was required to maintain the atomic positions of the cluster fixed.

The interaction energy (E_{int}) between oseltamivir and the Y-MOF cluster for both binding modes was calculated using equation 1:

$$E_{int} = (E_{cluster/oseltamivir}) - (E_{cluster} + E_{oseltamivir}) \qquad \text{(equation 1)}$$

where $E_{cluster/oseltamivir}$ is the energy of the optimized cluster/oseltamivir geometry, and $E_{cluster}$ and $E_{oseltamivir}$ are the single point energies of the individual components. The frontier molecular orbital and energy transport analysis for the preferential binding modes were explored further.

3. Experimental Section

3.1 Preparation of $[Y_2(L)_3(H_2O)_{1.5}(DMF)]_n$, namely Y-MOF.

A mixture of Yttrium chloride (15.1 mg, 0.05 mmol) and H_2L (15.4 mg, 0.05 mmol) was added into the mixture of DMF (4 mL) and H_2O (3 mL). The mixed solutions were sealed in a 25 mL Teflon reactor, after the reaction mixture had been heated at 120 °C for one day and then cooled to room temperature after 48 hours, colorless bulky crystals of **1** were isolated in 45 % yield based on H_2L ligand. Elemental analysis found (%) for $C_{54}H_{39}N_7O_{14.50}Y_2$: C 53.93 H 3.21 N 8.16; calcd: C 54.24, H 3.29, N 8.20. FT-IR data (cm^{-1}): 3651 (w), 3151(w), 1661 (m), 1611(m), 1583(m), 1527 (s), 1413(s), 1379(vs), 776(vs), 733 (vs).

3.2 Preparation of Y-MOF (1).

A mixture of Yttrium chloride (30.2 mg, 0.1 mmol), and H_2L (30.8 mg, 0.1 mmol) was added into the mixture of DMF (1 mL) and sodium acetate aqueous solution (5 mM, 0.5 mL). The mixed solutions were sealed in a 25 mL Teflon reactor, after the reaction mixture had been heated at 120 °C for one day and then cooled to room temperature naturally, colorless bulky crystals of Y-MOF (1). were isolated in 52 % yield based on H_2L ligand.

3.2 Preparation of Y-MOF (2).

A mixture of Yttrium chloride (30.2 mg, 0.1 mmol), and H_2L (30.8 mg, 0.1 mmol) was added into the mixture of DMF (1 mL) and sodium acetate aqueous solution (5 mM, 0.5 mL). The mixed solutions were sealed in a 25 mL Teflon reactor, after the reaction mixture had been heated at 120 °C for one day and then cooled to room temperature naturally, colorless bulky crystals of Y-MOF (2) were isolated in 52 % yield based on H_2L ligand.

3.3 Preparation of Y-MOF (3).

A mixture of Yttrium chloride (30.2 mg, 0.1 mmol), and H_2L (30.8 mg, 0.1 mmol) was added into the mixture of DMF (1 mL) and sodium acetate aqueous solution (10 mM, 0.5 mL). The mixed solutions were sealed in a 25 mL Teflon reactor, after the reaction mixture had been heated at 120 °C for one day and then cooled to room temperature naturally, colorless bulky crystals of Y-MOF (3) were isolated in 53 % yield based on H_2L ligand.

3.4 Preparation of COVID-19 effective drug solution

In the selective detection process, we prepared lopinavir, oseltamivir, ritonavir, chloroquire phosphate solution with 1 μM solution and DMSO as solvent, respectively.

3.5 Preparation of COVID-19 biomarker solution

In the selective detection process, the concentration of Y-MOF is 0.1 g/L, 960μL and the concentration of S2 is 1 μM, 40 μL, we prepared L-lactate Dehydrogenase (L-LDH), D-dimer, Creastinine, Interleukin-6, Interleukin-10 solution with 100 nM and PBS buffer (pH = 7.4) as solvent.

In the IL-6 titration process, we prepared IL-6 solution with 0.48 μM, PBS as solvent.

To get simulated human body fluid, we mixed creatine, L-LDH, D-dimer, Interleukin-10 into 5% human serum solution and the concentration of creatine is 133 μM, the concentration of L-LDH is 318 U/L, the concentration of D-dimmer is 1 μg/mL and IL-10 is 9.7 pg/mL.

The Sequence of S1:

5'-FAM- aagcttacct gccatgccag tacccccagg agaagattcc aaagatgtgg ccgccccaca-3'

The Sequence of S2:

5'-FAM-ttttttt aagcttacct gccatgccag tacccccagg agaagattcc aaagatgtgg ccgccccaca aaaaaaa-3'

The Sequence of S3 (a mutated aptamer for S2):

5'-FAM-tttttt aagcttaccg gccatgccag tacccaaagg agaagattcc aaagatttgg ccgcggcaca aaaaaaa-3'

Table S1. Crystallographic data and details of refinements for Y-MOF[a,b]

	Y-MOF
Formula	$C_{54}H_{39}N_7O_{14.50}Y_2$
M (g mol^{-1})	1195.74
Crystal system	Triclinic
Space group	P -1
a (Å)	9.941(3)
b (Å)	14.577(5)
c (Å)	18.928(6)
α (deg)	88.090(7)
β (deg)	77.833(7)
γ (deg)	74.266(6)
V (Å^3)	2580(14)
Z	2
F (000)	1212
ρ_{calc} (Mg m^{-3})	1.539
μ (mm^{-1})	2.314
data/restraints/parameters	10678 / 0 / 707
GOF on F^2	1.047
R_1[a] (I = 2σ(I))	0.0576
ωR_2[b] (all data)	0.1655

[a] $R_1 = \Sigma||F_o| - |F_c||/|F_o|$. [b] $\omega R_2 = [\Sigma w(|F_o|^2-|F_c^2|^2/w|F_o^2|^2]^{1/2}$.

Table S2. Selected bond lengths [Å] for Y-MOF.

Y-MOF			
Y1—O1^{i}	2.282 (3)	Y2—O10^{iv}	2.300 (3)
Y1—O3^{ii}	2.329 (4)	Y2—O8	2.302 (3)
Y1—O5	2.331 (3)	Y2—O7^{iv}	2.326 (3)
Y1—O2	2.359 (3)	Y2—O11^{v}	2.349 (4)
Y1—O6^{i}	2.365 (3)	Y2—O9	2.375 (3)
Y1—O13	2.377 (4)	Y2—O15	2.402 (4)
Y1—O4^{ii}	2.476 (4)	Y2—N4^{vi}	2.509 (4)
Y1—N2^{iii}	2.604 (4)	Y2—O12^{v}	2.644 (4)
Y1—O1	2.738 (4)	Y2—O10	2.779 (4)

Symmetry codes: (i) −x+2, −y+1, −z+1; (ii) x+1, y, z; (iii) x+1, y−1, z; (iv) −x+2, −y+1, −z; (v) x−1, y, z; (vi) −x+2, −y, −z.

Table S3. Selected angles [º] for Y-MOF.

Y-MOF			
$O1^{i}$—Y1—$O3^{ii}$	97.88 (13)	$O10^{iv}$—Y2—O8	74.08 (12)
$O1^{i}$—Y1—O5	81.79 (12)	$O10^{iv}$—Y2—$O7^{iv}$	78.00 (12)
$O3^{ii}$—Y1—O5	141.15 (13)	O8—Y2—$O7^{iv}$	132.06 (12)
$O1^{i}$—Y1—O2	124.59 (12)	$O10^{iv}$—Y2—$O11^{v}$	95.41 (14)
$O3^{ii}$—Y1—O2	127.30 (12)	O8—Y2—$O11^{v}$	78.42 (13)
O5—Y1—O2	80.38 (13)	$O7^{iv}$—Y2—$O11^{v}$	142.95 (13)
$O1^{i}$—Y1—$O6^{i}$	73.03 (12)	$O10^{iv}$—Y2—O9	124.13 (12)
$O3^{ii}$—Y1—$O6^{i}$	82.50 (13)	O8—Y2—O9	78.02 (13)
O5—Y1—$O6^{i}$	132.81 (12)	$O7^{iv}$—Y2—O9	86.72 (13)
O2—Y1—$O6^{i}$	82.10 (13)	$O11^{v}$—Y2—O9	124.89 (13)
$O1^{i}$—Y1—O13	139.76 (13)	$O10^{iv}$—Y2—O15	76.16 (13)
$O3^{ii}$—Y1—O13	84.14 (14)	O8—Y2—O15	134.62 (14)
O5—Y1—O13	72.69 (12)	$O7^{iv}$—Y2—O15	71.90 (13)
O2—Y1—O13	81.63 (13)	$O11^{v}$—Y2—O15	71.13 (14)
$O6^{i}$—Y1—O13	146.20 (12)	O9—Y2—O15	147.32 (14)
$O1^{i}$—Y1—$O4^{ii}$	139.85 (13)	$O10^{iv}$—Y2—$N4^{vi}$	147.12 (13)
$O3^{ii}$—Y1—$O4^{ii}$	54.02 (12)	O8—Y2—$N4^{vi}$	138.75 (13)
O5—Y1—$O4^{ii}$	138.34 (12)	$O7^{iv}$—Y2—$N4^{vi}$	77.20 (13)
O2—Y1—$O4^{ii}$	73.33 (12)	$O11^{v}$—Y2—$N4^{vi}$	91.54 (15)

O6^{i}—Y1—O4^{ii}	75.04 (12)	O9—Y2—N4^{vi}	75.47 (14)
O13—Y1—O4^{ii}	71.98 (13)	O15—Y2—N4^{vi}	75.84 (14)
O1^{i}—Y1—N2^{iii}	71.23 (13)	O10^{iv}—Y2—O12^{v}	135.42 (12)
O3^{ii}—Y1—N2^{iii}	71.26 (13)	O8—Y2—O12^{v}	70.87 (12)
O5—Y1—N2^{iii}	71.98 (13)	O7^{iv}—Y2—O12^{v}	146.58 (12)
O2—Y1—N2^{iii}	145.94 (14)	O11^{v}—Y2—O12^{v}	51.36 (13)
O6^{i}—Y1—N2^{iii}	131.53 (14)	O9—Y2—O12^{v}	73.94 (12)
O13—Y1—N2^{iii}	71.54 (13)	O15—Y2—O12^{v}	111.08 (13)
O4^{ii}—Y1—N2^{iii}	115.75 (13)	N4^{vi}—Y2—O12^{v}	71.75 (13)
O1^{i}—Y1—O1	74.96 (12)	O10^{iv}—Y2—O10	75.12 (12)
O3^{ii}—Y1—O1	153.55 (12)	O8—Y2—O10	67.41 (11)
O5—Y1—O1	64.09 (11)	O7^{iv}—Y2—O10	68.19 (11)
O2—Y1—O1	50.07 (10)	O11^{v}—Y2—O10	145.83 (13)
O6^{i}—Y1—O1	71.06 (12)	O9—Y2—O10	49.49 (10)
O13—Y1—O1	117.99 (12)	O15—Y2—O10	134.54 (12)
O4^{ii}—Y1—O1	116.26 (11)	N4^{vi}—Y2—O10	114.39 (13)
N2^{iii}—Y1—O1	127.30 (12)	O12^{v}—Y2—O10	114.16 (11)

Symmetry codes: (i) −x+2, −y+1, −z+1; (ii) x+1, y, z; (iii) x+1, y−1, z; (iv) −x+2, −y+1, −z; (v) x−1, y, z; (vi) −x+2, −y, −z.

Table S4. Hydrogen bonds for Y-MOF (1) [Å and °].

D-H···A	d(D-H)	d(H···A)	d(D···A)	<(DHA)
C(32)-H(32)...O(14)[a]	0.93	2.44	3.339(12)	164
C(33)-H(33)...O(2)[b]	0.93	2.42	3.158(7)	136

Symmetry codes: (a) -x+1, -y, -z ; (b) x, y-1, z

Table S5. Average size and standard deviation measured by scanning electron microscopy (SEM) and dynamic light scattering (DLS, number mean value is reported)

Sample	SEM	Standard deviation	DLS
Y-MOF (1)	106 nm	20.7 nm	108 nm
Y-MOF (2)	109 nm	23.7 nm	115 nm
Y-MOF (3)	118 nm	21.5 nm	122 nm

Table S6. The average fluorescence lifetime of Y-MOF after adding different concentrations of DNA sequence S2 (excitation at 260 nm).

Sample Name	Wavelength/nm	Lifetime/ns
Y-MOF	370	1.57
Y-MOF + S2 (0.1 μM)	370	1.55
Y-MOF + S2 (0.2 μM)	370	1.52
Y-MOF + S2 (0.3 μM)	370	1.48
Y-MOF + S2 (0.4 μM)	370	1.46

Table S7. Results of IL-6 detection in human blood (n=3)

Sample	Added/nM	Found/nM	Recovery/%	RSD/%
1	0	Not detected		
2	0.96	0.947	98.6	1.02
3	1.92	1.966	102.4	1.12
4	2.88	2.926	101.6	0.52
5	3.84	3.756	97.8	1.87
6	4.8	4.810	100.2	1.32
7	5.76	5.846	101.5	0.67
8	6.72	6.673	99.3	1.04
9	7.68	7.918	103.1	0.84
10	8.64	8.856	102.5	1.33

Table S8. The average fluorescence lifetime of Y-MOF@S2 after adding different concentration of IL-6 (excitation at 260 nm).

Sample Name	Wavelength/nm	Lifetime/ns
Y-MOF @ S2 + IL-6	370	1.43
Y-MOF @ S2 + IL-6 (4.8 nM)	370	1.41
Y-MOF @ S2 + IL-6 (7.2 nM)	370	1.40
Y-MOF @ S2 + IL-6 (9.6 nM)	370	1.38

Table S9. Results of Oseltamivir detection in human blood (n=3)

Sample	Added/nM	Found/nM	Recovery/%	RSD/%
1	0			
2	0.02	0.020	101.4	0.712
3	0.06	0.059	98.2	0.689
4	0.08	0.083	103.2	0.547
5	0.1	0.104	104.3	0.583
6	0.12	0.128	106.8	0.428
7	0.14	0.142	101.3	0.764
8	0.16	0.154	96.4	0.857
9	0.18	0.188	104.2	0.662
10	0.2	0.203	101.7	0.785
11	0.22	0.213	96.9	0.403
12	0.24	0.241	100.6	0.659
13	0.26	0.261	100.4	0.632
14	0.28	0.273	97.5	0.723

Table S10. The average fluorescence lifetime of Y-MOF@S2@IL-6 after adding different concentrations of Oseltamivir (excitation at 260 nm).

**Sample Name**	**Wavelength/nm**	**Lifetime/ns**
Y-MOF @ S2 @ IL-6	370	1.38
Y-MOF @ S2 @ IL-6 + Oseltamivir (0.1 μM)	370	1.39
Y-MOF @ S2 @ IL-6 + Oseltamivir (0.2 μM)	370	1.38
Y-MOF @ S2 @ IL-6 + Oseltamivir (0.3 μM)	370	1.39
Y-MOF @ S2 @ IL-6 + Oseltamivir (0.4 μM)	370	1.40
Y-MOF @ S2 @ IL-6 + Oseltamivir (0.5 μM)	370	1.39

Table S11. The average fluorescence lifetime of Y-MOF@S2 after adding different concentrations of IL-6 (excitation at 380 nm).

Sample Name	Wavelength/nm	Lifetime/ns
Y-MOF (3)@S2 + IL6	514	2.89
Y-MOF (3)@S2 + IL6 (4.8 nM)	514	2.89
Y-MOF (3)@S2 + IL6 (9.6 nM)	514	2.9

Table S12. The average fluorescence lifetime of Y-MOF@S2 after adding different concentrations of Oseltamivir (excitation at 380 nm).

Sample Name	Wavelength/nm	Lifetime/ns
Y-MOF (3)@S2@IL6 + Oseltamivir (0)	514	2.52
Y-MOF (3)@S2@IL6 + Oseltamivir (0.3 μm)	514	2.54

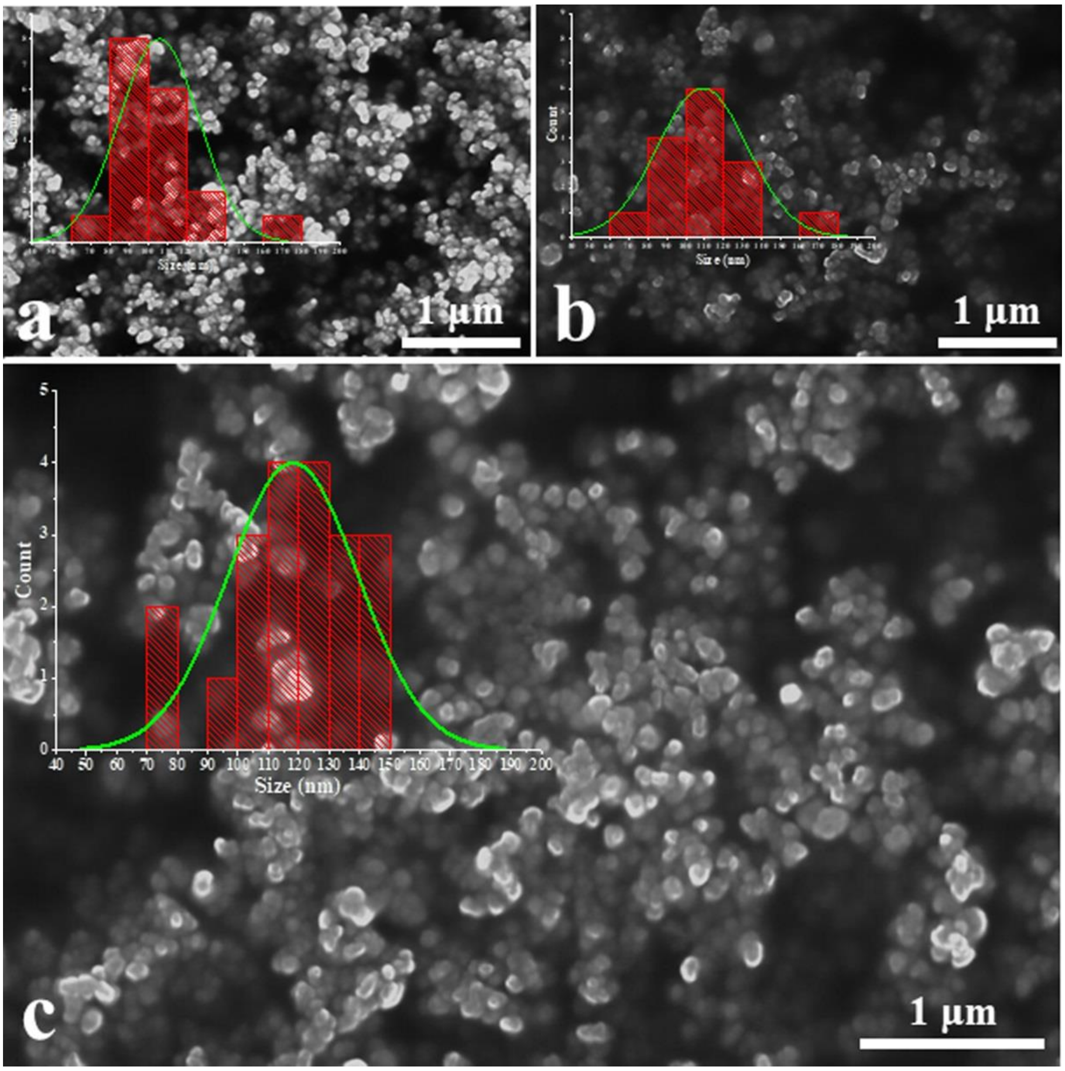

Figure S1. SEM images of (a) Y-MOF(1), the average particle size is 106 nm; (b) Y-MOF (2), the average particle size is 109 nm; (c) Y-MOF(3), the average particle size is 118 nm.

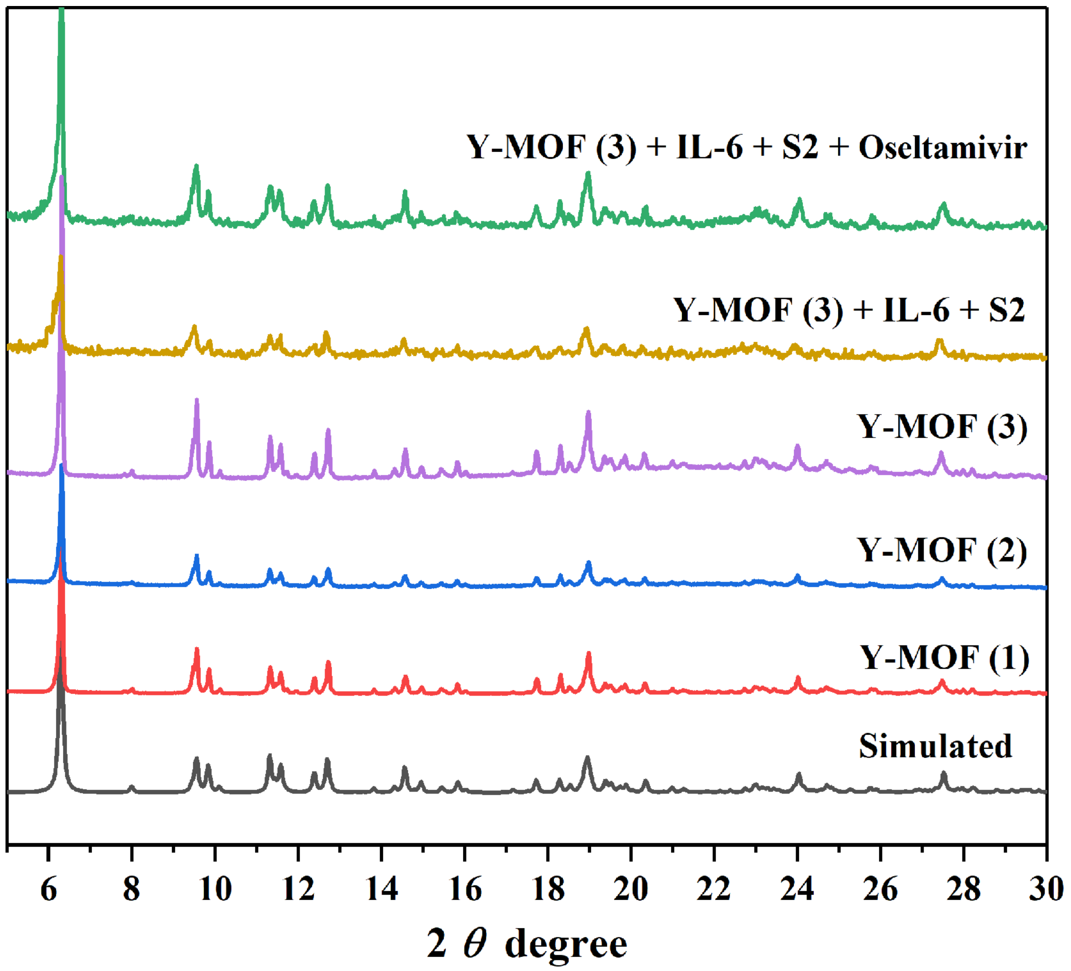

Figure S2. PXRD patterns of Y-MOF in different synthetic conditions.

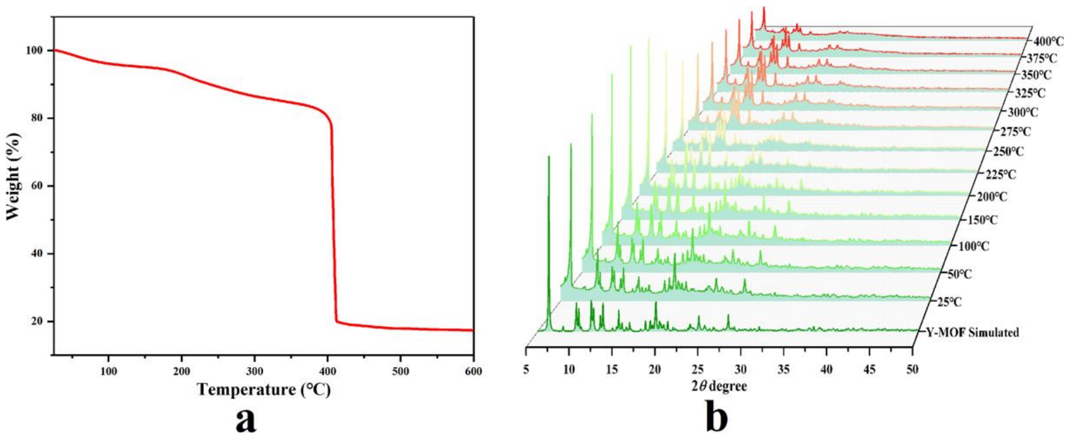

Figure S3. (a) TG analysis of Y-MOF; (b) Variation temperature XRD patterns of Y-MOF.

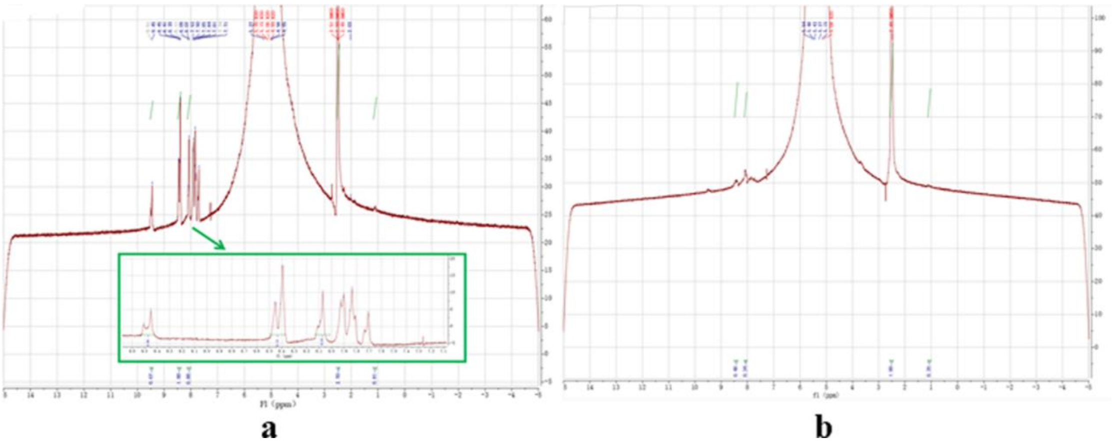

Figure S4. ^{1}HNMR spectra of digested (a) MOF **2** and (b) MOF **3**.

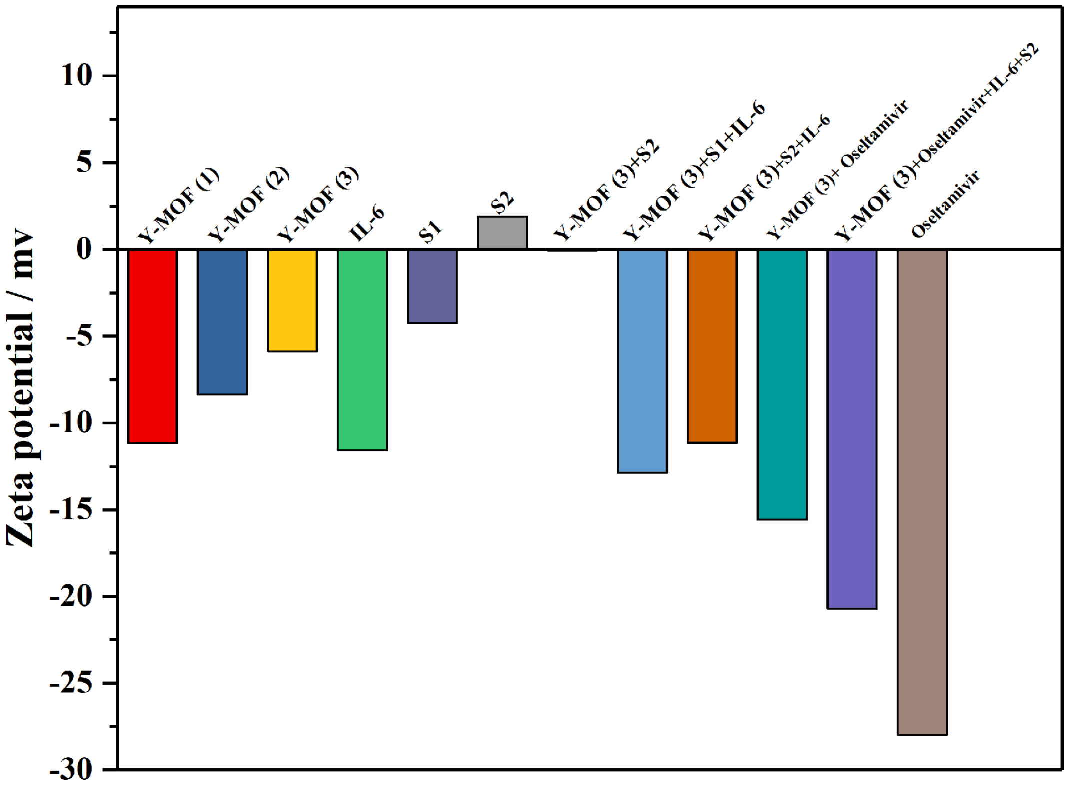

Figure S5. Zeta potential of Y-MOF, DNA sequence and Oseltamivir under different conditions.

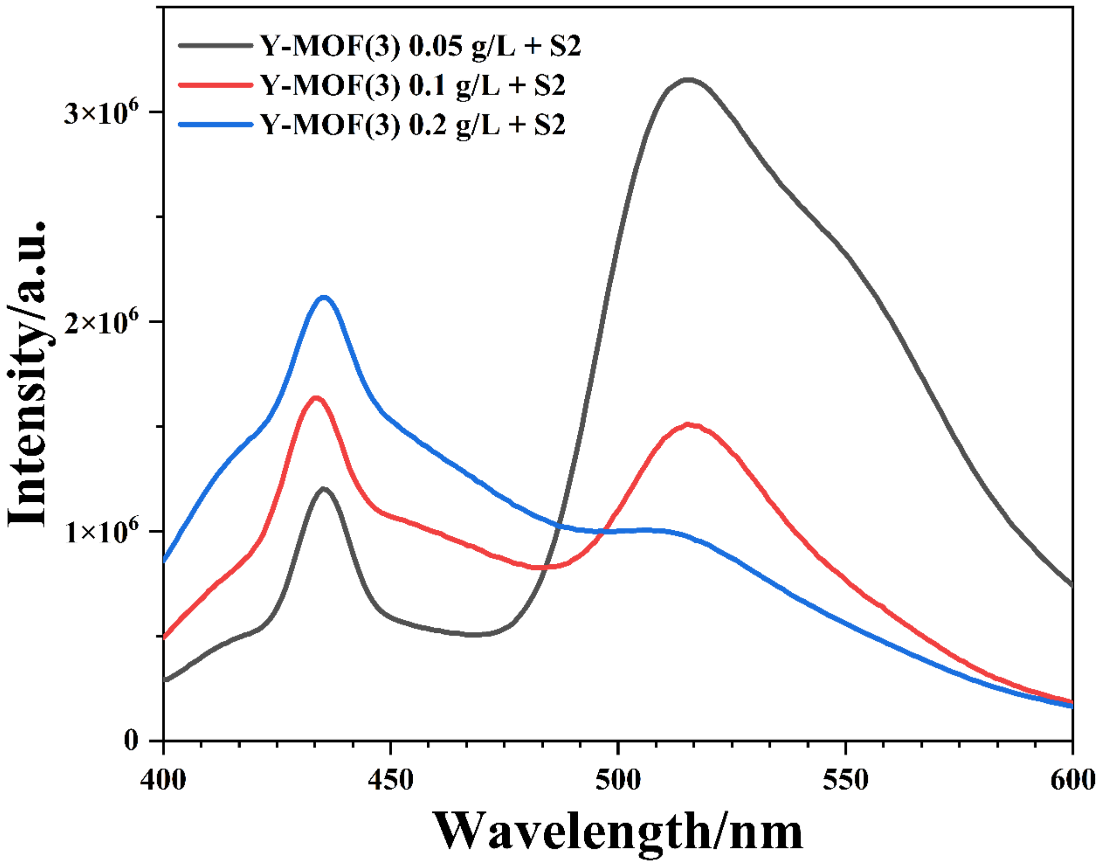

Figure S6. Fluorescence spectra of the mixture of Y-MOF (0.05 g/L), Y-MOF (0.1 g/L), Y-MOF (0.2 g/L) with S2 (1 μM) respectively (excitation at 380 nm).

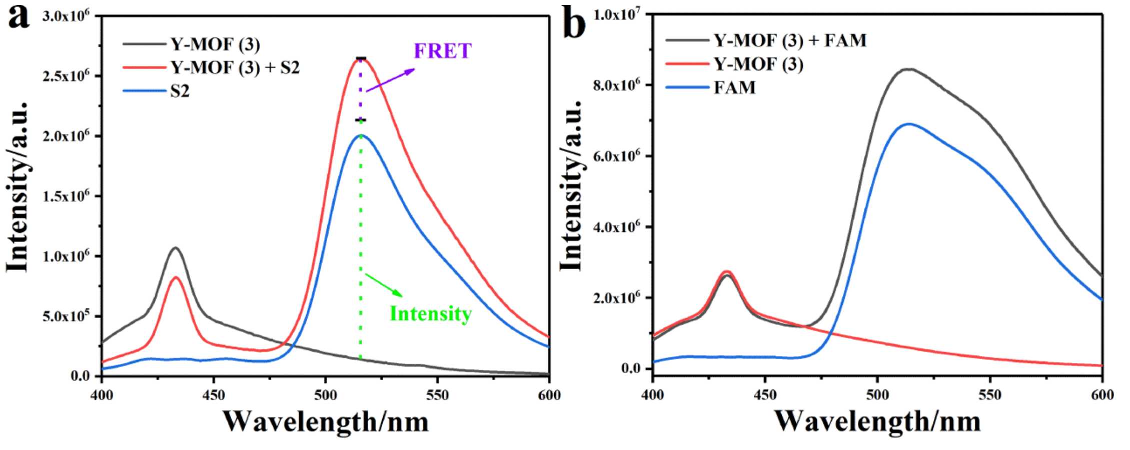

Figure S7. (a) Fluorescence spectra of Y-MOF (0.05 g/L), S2 (1 μM) and the mixture of S2 (1 μM) and Y-MOF (0.05 g/L) (excitation at 380 nm); (b) Fluorescence spectra of Y-MOF (0.1 g/L), FAM (1 μM) and the mixture of Y-MOF (0.1 g/L), FAM (1 μM) (excitation at 380 nm).

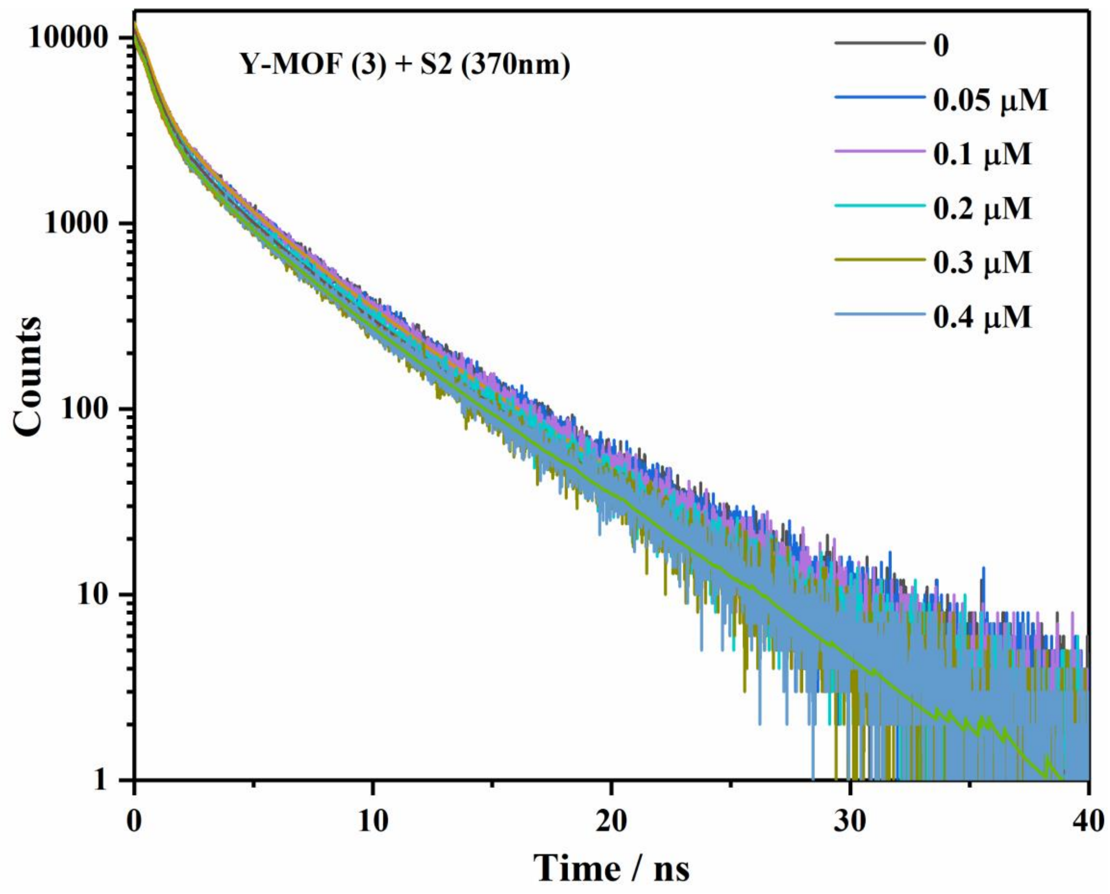

Figure S8. Fluorescence decay curves at 370 nm of Y-MOF (3) after adding different concentrations of DNA sequence S2 (excitation position at 260 nm).

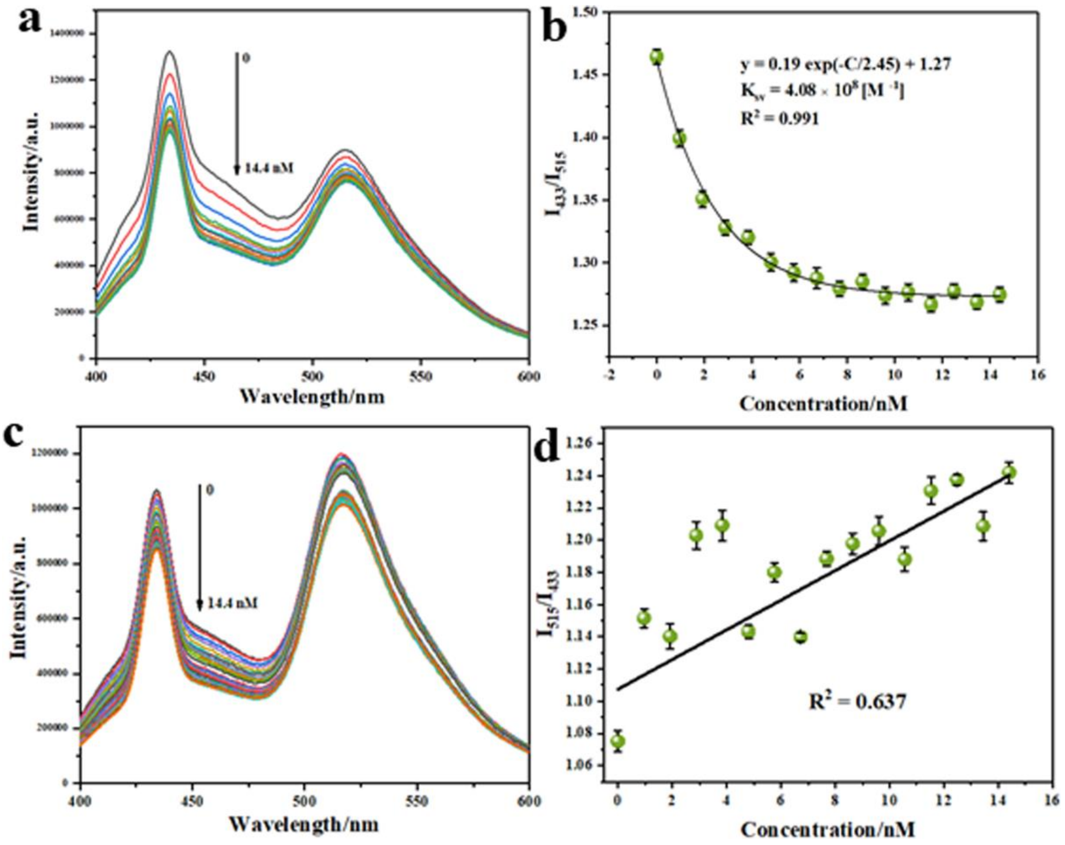

Figure S9. (a) Fluorescence spectra of **Y-MOF(2)@S2** after adding various concentrations of IL-6 (excitation at 380 nm); (b) Non-linear relationship between concentration of IL-6 and the fluorescence intensity ratio of **Y-MOF(2)@S2**; (c) Fluorescence spectra of **Y-MOF(1)@S2** after adding various concentrations of IL-6 (excitation at 380 nm); (d) Linear relationship between the concentration of IL-6 and the fluorescence intensity ratio of **Y-MOF(1)@S2**.

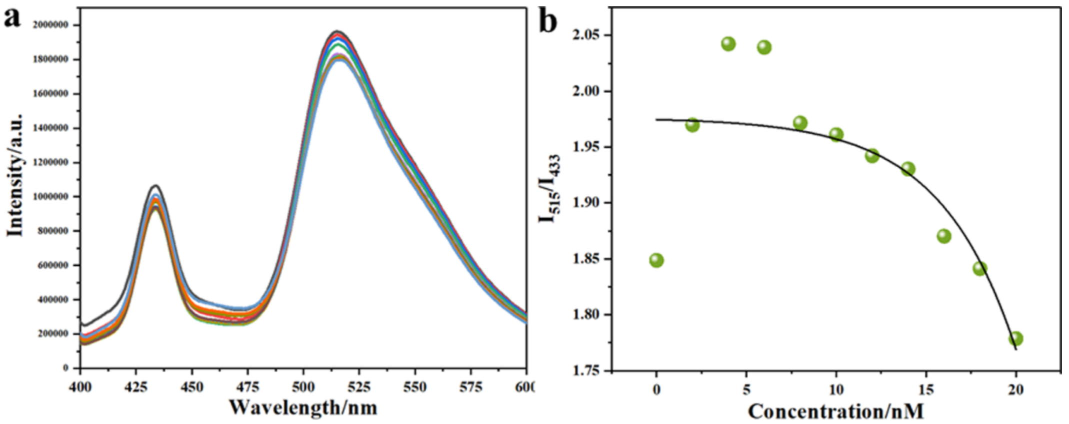

Figure. S10: (a) Fluorescence spectra of **Y-MOF@S1** adding various concentrations of IL-6 which excited at 380 nm. (b) Non-linear relationship between concentration of IL-6 and the fluorescence intensity ratio of **Y-MOF@S1**.

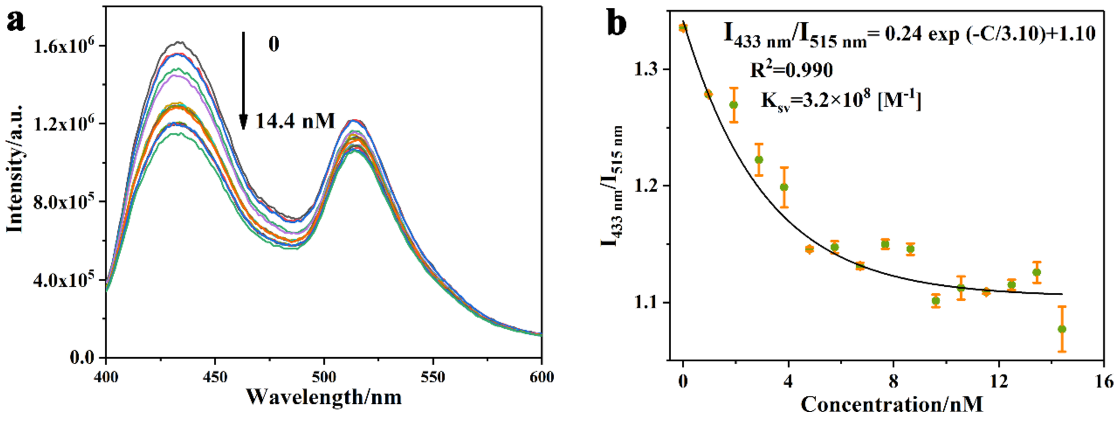

Figure S11. (a) Fluorescence spectra of **Y-MOF@S3** adding various concentrations of IL-6 (excitation at 380 nm). (b) Non-linear relationship between concentration of IL-6 and the fluorescence intensity ratio of **Y-MOF@S3**.

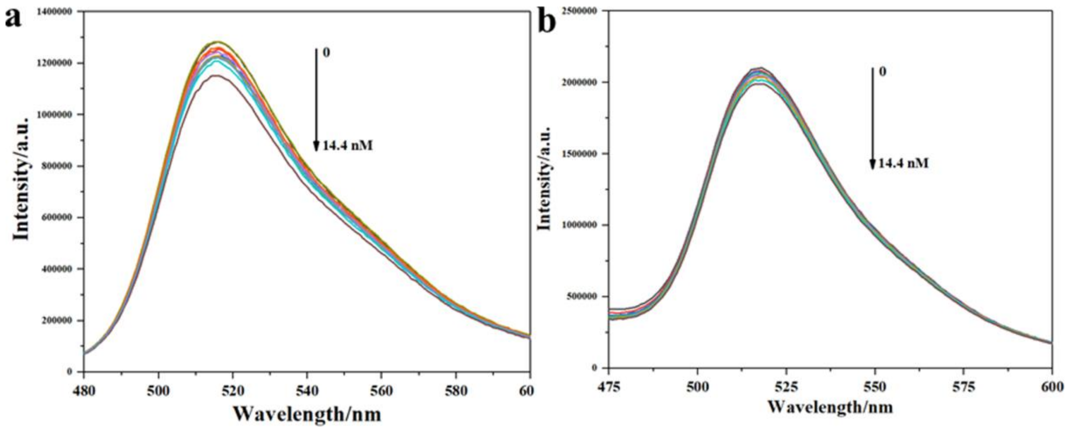

Figure S12. (a) Fluorescence spectra of S2 (1 μmol) adding various concentrations of IL-6 (excitation at 380 nm); (b) Fluorescence spectra of S1 (1 μmol) after adding various concentrations of IL-6 (excitation at 380 nm).

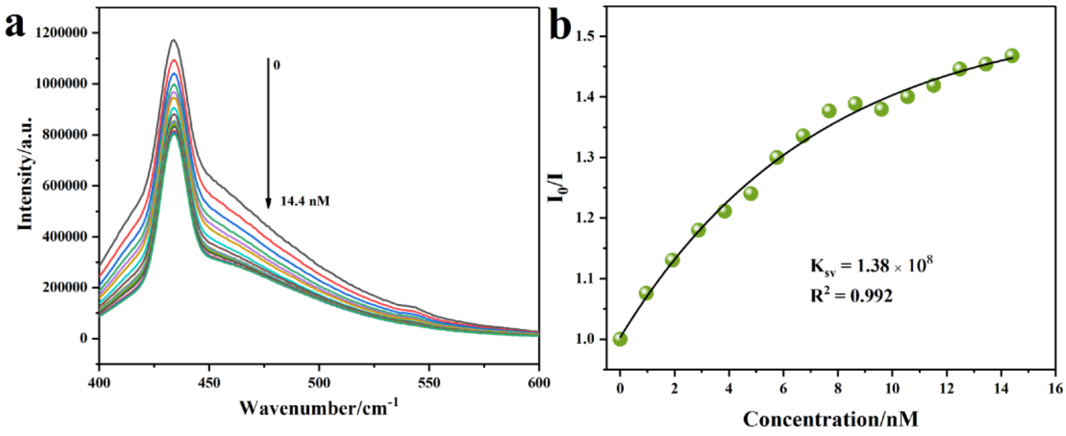

Figure S13 (a) Fluorescence spectra of **Y-MOF (3)** (0.1 g/L) after adding various concentrations

of IL-6 which excited at 380 nm. (b) Non-linear relationship between concentration of IL-6 and the fluorescence intensity ratio of **Y-MOF (3)**.

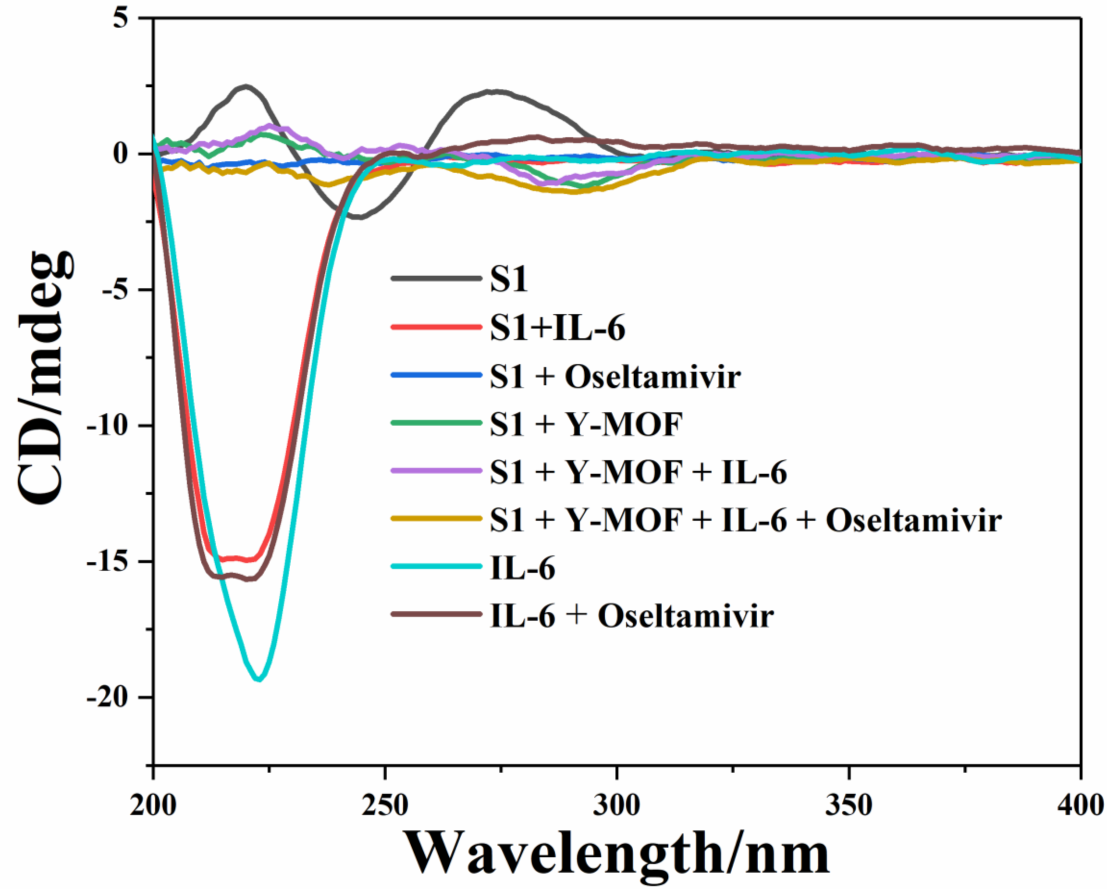

Figure S14. CD spectrum of IL-6 and S1 in different solutions.

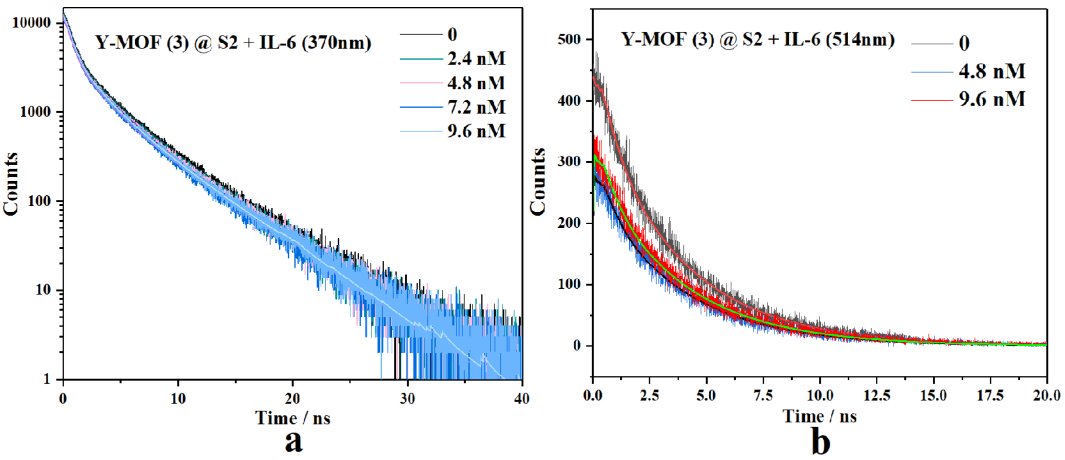

Figure S15. (a) Fluorescence decay curves at 370 nm of **Y-MOF(2)@S2** after adding various concentrations of IL-6 (excitation at 260 nm); (b) Fluorescence decay curves at 514 nm of **Y-MOF(2)@S2** after adding various concentrations of IL-6 (excitation at 380 nm).

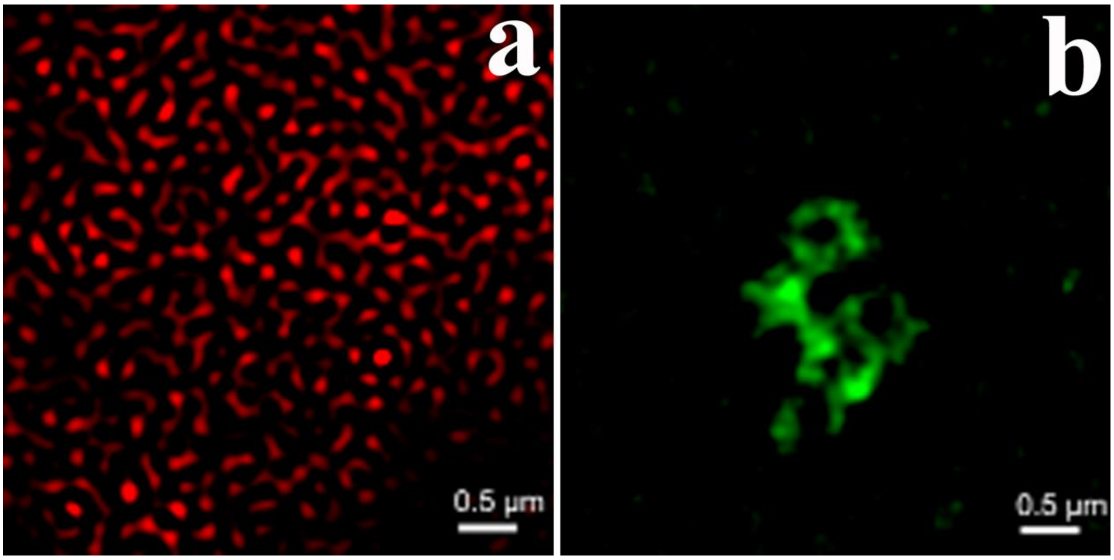

Figure S16. SIM images of (a)Y-MOF, (b) S2.

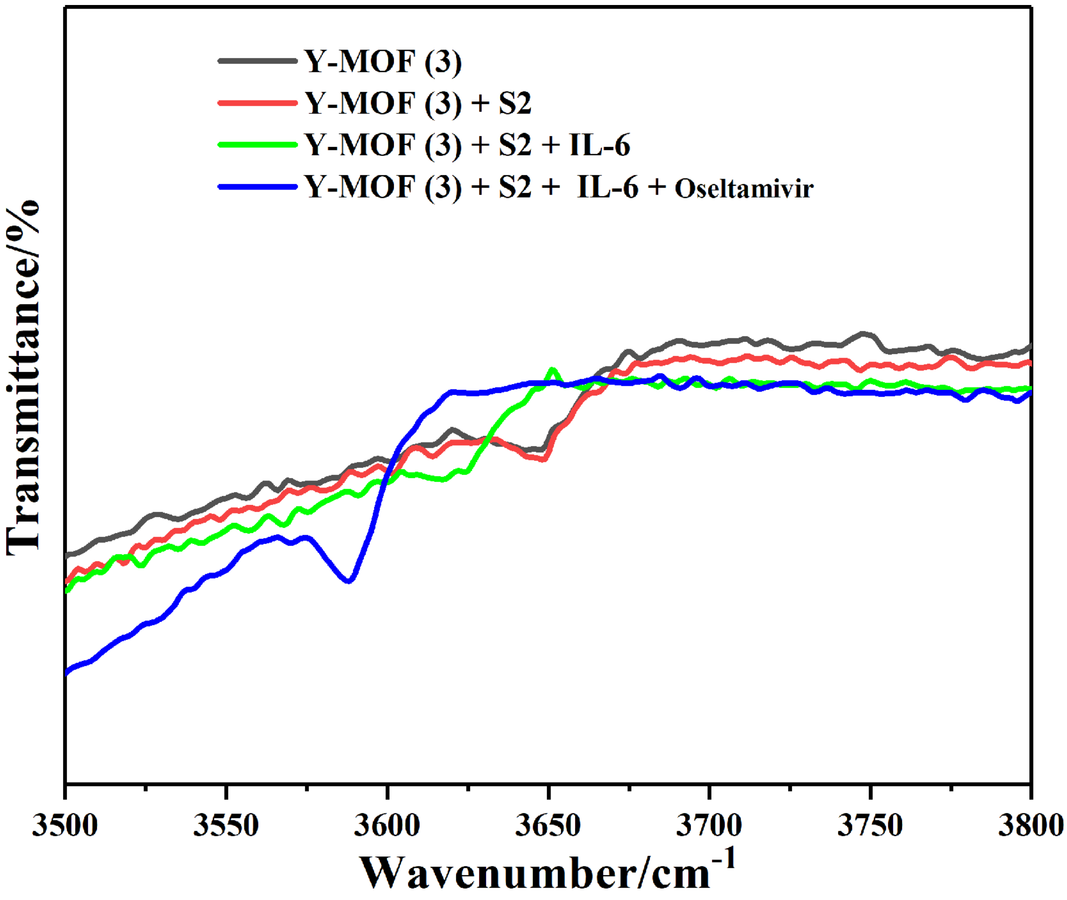

Figure S17. FT-IR spectrum of Y-MOF (3) under different conditions.

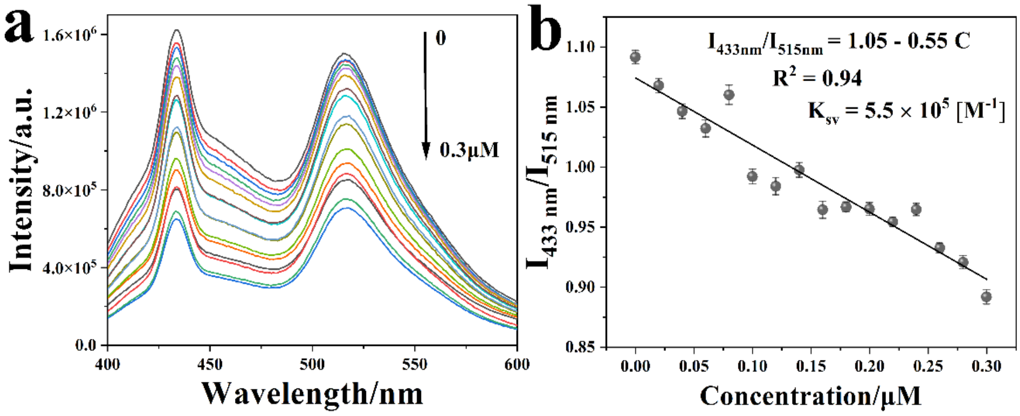

Figure S18. (a) Fluorescence spectra of **Y-MOF@S2** after adding various concentration of Oseltamivir under excitation at 380 nm in human serum (5 μL, water as solvent). (b) Linear relationship between concentration of Oseltamivir and the fluorescence intensity ratio of **Y-MOF@S2**.

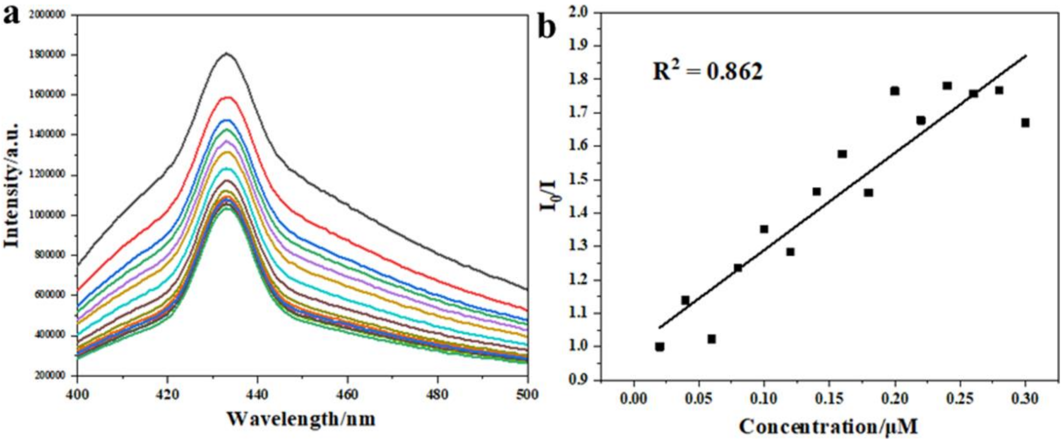

Figure S19: (a) Fluorescence spectra of Y-MOF after adding Oseltamivir at different concentrations (from 0 to 0.30 μM, excitation at 380 nm); (b) Linear relationship between the concentration of oseltamivir and fluorescence intensity ratio of Y-MOF.

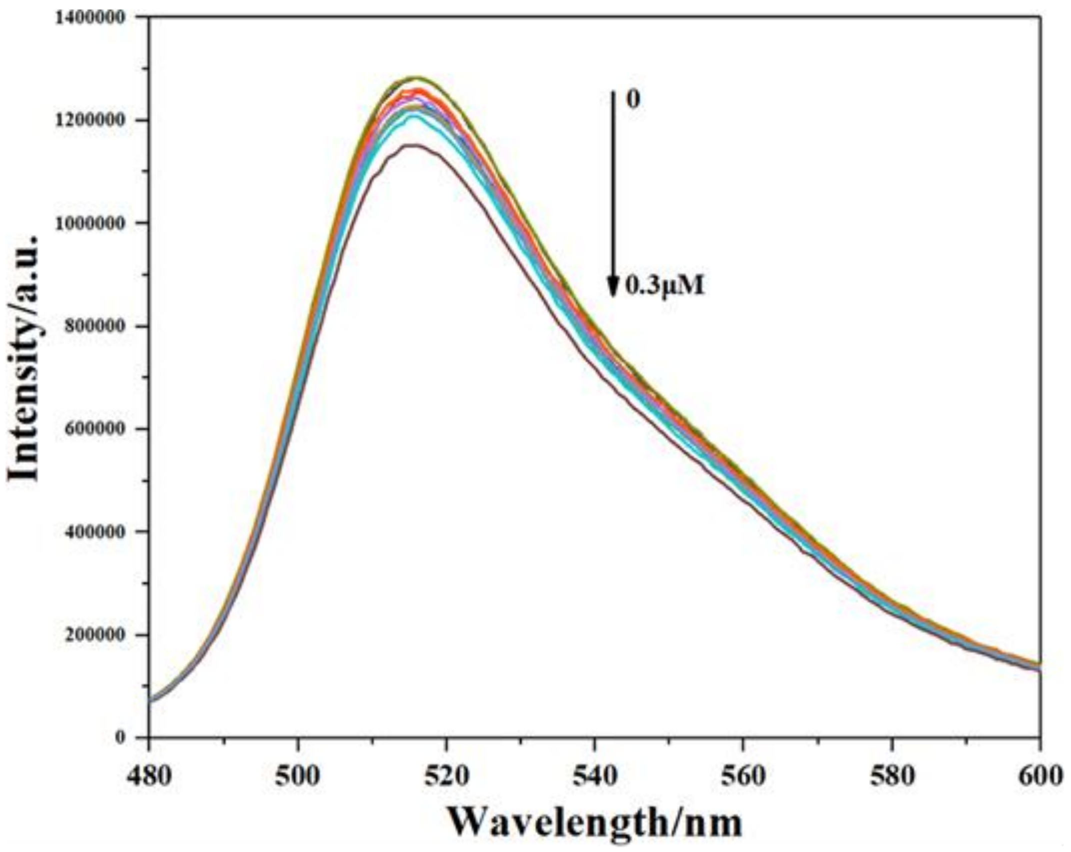

Figure S20. Fluorescence spectra of S2 with IL-6 after adding Oseltamivir at different concentrations (from 0-0.3 μM).

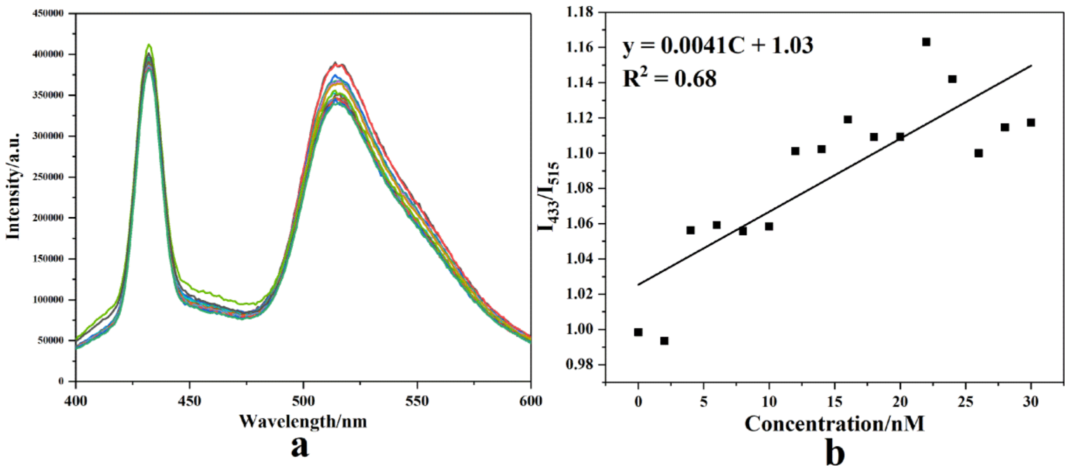

Figure S21. (a) Fluorescence spectra of **Y-MOF(3)@S2** in human serum after adding different concentration of Oseltamivir; (b) Linear relationship between the concentration of Oseltamivir and the fluorescence intensity ratio of **Y-MOF(3)@S2**.

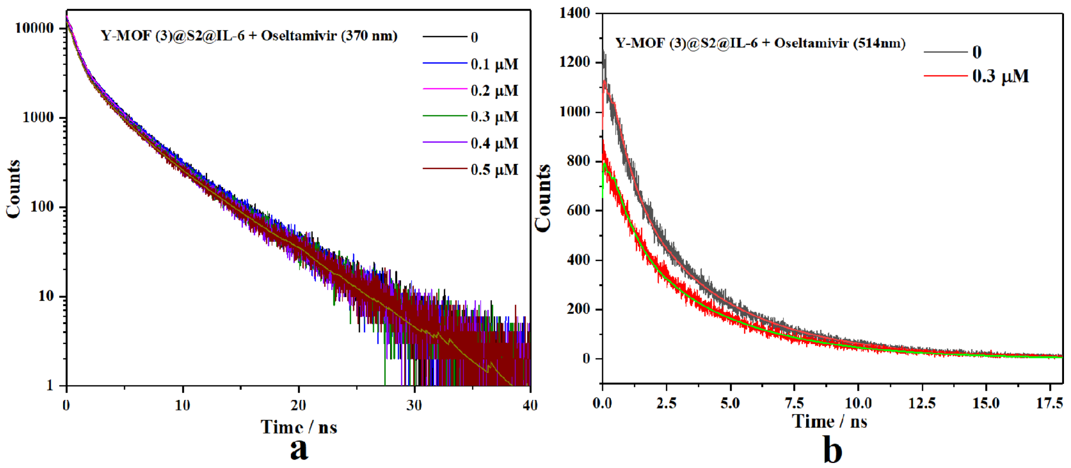

Figure S22. (a) Fluorescence decay curves of **Y-MOF(3)@S2@IL-6** recorded at 370 nm after adding different concentrations of Oseltamivir (excitation at 260 nm); (b) Fluorescence decay curves of **Y-MOF(2)@S2@IL-6** recorded at 514 nm after adding different concentrations of Oseltamivir (excitation at 380 nm).

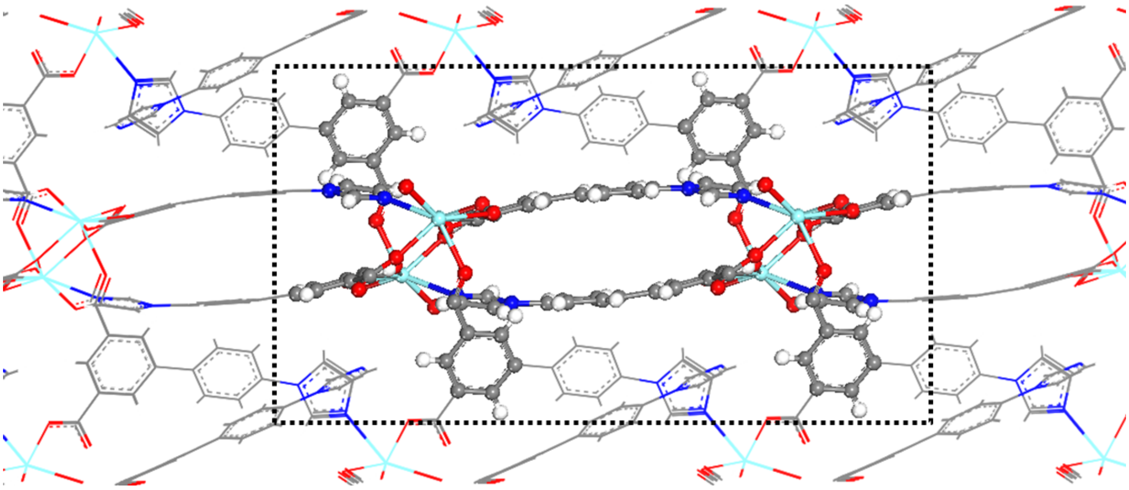

Figure S23. Illustration of the cluster model (ball and stick) cleaved from the periodic crystal structure of the Y-MOF (line).

`

References

[1] Vakili, M., Romano, E., Darugar, V., Brandán, S. A., J Mol Model 27, 357, 2021

[2] Frisch, M. J.; et al. Gaussian 09, Revision D.01; Gaussian, Inc.: Wallingford, CT, 2013

[3] A. D. Becke, J. Chem. Phys., 1993, 98, 5648-5652.

[4] C. Lee, W. Yang and R. G. Parr, Phys. Rev. B, 1988, 37, 785-789.

[5] Grimme, S.; Antony, J.; Ehrlich, S.; Krieg, H., J. Chem. Phys 2010, 132, 154104.

[6] Grimme, S.; Ehrlich, S.; Goerigk, L., J. Comput. Chem. 2011, 32, 1456-1465.

[7] Petersson, G. A.; Bennett, A.; Tensfeldt, T. G.; Al-Laham, M. A.; Shirley, W. A.; Mantzaris, J., J. Chem. Phys. 1988, 89, 2193−2218

[8] M. Dolg, H. Stoll and H. Preuss, J. Chem. Phys., 1989, 90, 1730–1734.

[9] X. Cao and M. Dolg, J. Mol. Struct.: THEOCHEM, 2002, 581, 139–147.

[10] Rad, Ali Shokuhi, et al. Spectrochim. Acta A. 2021, 247, 119082.
